# A validated sequence difference ratio framework distinguishes intrahost evolution from superinfection in *Helicobacter pylori* mixed infections

**DOI:** 10.3389/fmicb.2026.1948969

**Published:** 2026-10-06

**Authors:** Haiying Gu, Zhouhong Shi, Weiqin Chen, Weiwei Ye, Cheng Zhang, Airu Li

**Affiliations:** 1 Health Science Center, Ningbo University, Ningbo, Zhejiang, China; 2 Laboratory of Helicobacter Pylori Section, Professional Committee of Clinical Microbiology, Chinese Society for Microbiology, Ningbo University, Ningbo, Zhejiang, China; 3 Department of Gastrology, Ninghai First Hospital, Ningbo, Zhejiang, China

**Keywords:** *Helicobacter pylori*, mixed infection, mutation, recombination, sequence difference ratio, superinfection

## Abstract

**Background:**

The inability to quantitatively distinguish endogenous within-host evolution (mutation, homologous recombination, and heterologous recombination) from superinfection as the origin of intrahost *Helicobacter pylori* genetic diversity remains a critical bottleneck constraining precision therapeutics.

**Methods:**

We developed a novel gene-based sequence difference ratio (SDR) framework utilizing single-nucleotide polymorphism counts and percentile-derived regional reference ranges to quantitatively classify the origins of mixed infections, including homologous and heterologous recombination (interspecies horizontal gene transfer). A dual-hospital cohort (34 patients, 114 flagellar isolates; 28 patients, 152 housekeeping isolates) was enrolled after excluding those with recent antibiotic or acid-suppressant exposure. Isolates were recovered using a patented single-colony culture system (US 18/278,425; EP 21927415.6) to eliminate conventional streaking bias. The model was validated using phylogenetic analysis, hierarchical clustering, and geographical divergence assessment.

**Results:**

The SDR framework achieved the first dedicated quantitative classification of five distinct *H. pylori* mixed infection mechanisms: mutation, homologous recombination, heterologous recombination, combined mutation–homologous recombination, and superinfection. Flagellar gene profiling identified a 51.5% mixed infection rate (17/33), with mutations as the dominant pattern (82.4%, 14/17); recombination involved the conserved flagellar genes *flgE* and *flaA*. Housekeeping gene analysis yielded a 14.8% superinfection rate (4/27), including the first documented triple-strain case; recombination involved the conserved housekeeping genes *atpA* and *yphC*. Strain H42 exhibited heterologous recombination in *trpC* (SDR 23.89–25.66%), providing the first nucleotide-resolution evidence of cross-species genetic exchange. Convergent mutations in *fliD* and *mutY* indicated niche-specific selective pressure. SDR thresholds were strongly correlated with evolutionary distance.

**Conclusion:**

We report the first validated quantitative framework for distinguishing intrahost microbial evolution (mutation, homologous recombination, and heterologous recombination) from superinfection in *H. pylori*. These findings challenge the long-standing single-strain therapeutic paradigm, and this scalable approach lays a universal foundation for the precise management of infectious diseases.

## 1 Introduction

Chronic microbial infections often harbor complex intraspecific strain diversity driven by three key evolutionary processes: mutation (Lundin et al., 2005; Jackson et al., 2020; Raymond et al., 2004), recombination (Raymond et al., 2004), and acquisition of unrelated strains via superinfection (Salama et al., 2007). A major challenge in clinical microbial genomics is the ability to quantitatively discriminate between distinct evolutionary pathways at high nucleotide resolution (Mendoza-Elizalde et al., 2019; Vazirzadeh et al., 2022). Conventional analytical methods fail to resolve the detailed characteristics of bacterial genetic variations within individual hosts. In particular, they cannot differentiate between the origins of the observed genomic diversity, including mutations, recombination, heterologous recombination, and convergent mutations. Likewise, these approaches cannot discriminate between within-host bacterial evolution and superinfection, which hinders the full elucidation of infection progression, bacterial persistence, and the molecular mechanisms underlying recurrent therapeutic failure.

*Helicobacter pylori*, a globally prevalent gastrointestinal pathogen transmitted predominantly within households (Garg et al., 2006), is a major cause of peptic ulcers and gastric cancer (Ansari and Yamaoka, 2020; Chmiela and Kupcinskas, 2019), and frequently exhibits mixed infections of genetically distinct strains (Kekic et al., 2024; Ben Mansour et al., 2016; Lai et al., 2016; Contreras et al., 2014). The reported mixed infection rates vary widely (Kekic et al., 2024; Ben Mansour et al., 2016; Lai et al., 2016; Zhang et al., 2023; Boyanova et al., 2023); however, the evolutionary drivers, particularly superinfections, remain poorly defined. Robust evidence for *H. pylori* superinfection is scarce compared with that for pathogens such as *Mycobacterium tuberculosis* (Kontsevaya et al., 2017), partly because of inconsistent definitions of “unrelated strains” and a lack of tools to resolve these mechanisms quantitatively. Unlike previous studies that defined unrelated strains solely using laboratory-based criteria (e.g., sequencing or electrophoresis patterns) (Mendoza-Elizalde et al., 2019; Zhang et al., 2023; Raaf et al., 2017; Carroll et al., 2004), this study adopted an epidemiology-based definition, considering strains as unrelated in the absence of epidemiological linkage. Based on these criteria, *H. pylori* isolates from randomly screened hosts were classified as epidemiologically unrelated.

Current genotyping methods, including multilocus sequence typing (MLST) (Mendoza-Elizalde et al., 2019; Zhang et al., 2023; Raaf et al., 2017; Secka et al., 2014) and whole-genome sequencing (Wilkinson et al., 2022; Didelot et al., 2013; Cao et al., 2015), critically depend on the culture-based recovery of single-colony isolates from gastrointestinal mucosal specimens. While useful for profiling strain diversity, these approaches lack adequate quantitative power for differentiating mutations and recombination and fail to reliably detect superinfection. Furthermore, flagellar genes, which are critical for *H. pylori* colonization and virulence (Gu, 2017; Aihara et al., 2014), remain underutilized in mixed infection studies, representing an additional gap in current research. Critically, Falush et al. (2001) sequentially collected *H. pylori* isolates from two clinical trials in Louisiana and Colombia rather than from patients with verified single-host mixed infections. Consequently, the genomic variations detected in their dataset could not be unambiguously assigned to *de novo* point mutations, homologous recombination, or superinfection-mediated strain displacement because their Bayesian model failed to distinguish between these distinct evolutionary and epidemiological scenarios.

To address these critical gaps, we developed a sequence difference ratio (SDR) framework that integrates epidemiological definitions, multigene sequencing, and phylogenetic validation. We hypothesized that the SDR framework could reliably discriminate between mutations, recombination, heterologous recombination, and superinfection; flagellar and housekeeping genes would exhibit distinct evolutionary patterns; and superinfection is a prevalent driver of *H. pylori* within-host diversity. Our primary aims were to establish gene-specific SDR reference ranges to define these core evolutionary mechanisms, characterize the dynamics of *H. pylori* superinfection, and create a generalizable tool for tracking the origins of within-host strain diversity.

## 2 Materials and methods

### 2.1 Study design and participants

*Helicobacter pylori* isolation, culture, and phenotypic drug susceptibility testing were routinely performed at the Affiliated Hospital of Ningbo University School of Medicine and Ninghai First Hospital. This two-hospital methodological study analyzed *H. pylori* isolates from 62 randomly selected patients at these institutions in Ningbo, China. Ethical approval for this study was granted by the Medical Research Committee of Ningbo University School of Medicine (approval number: NBU-2021-129), and written informed consent was obtained from all participants. The methodology is protected under PCT patent application PCT/CN2025/113728. The study analyzed two separate patient groups: 34 patients contributed 114 isolates for flagellar gene analysis (F group), and 28 patients contributed 152 isolates for housekeeping gene analysis (H group). *Helicobacter pylori* ATCC 700824 served as the control strain (Zhang et al., 2023). Exclusion criteria were recent use of antibiotics, acid suppressants, nonsteroidal anti-inflammatory drugs, or bismuth compounds. Biopsies from the antrum, corpus, and duodenal bulb were obtained during endoscopy, placed into a commercial transport medium (Gu’s Kit for Preservation of *Helicobacter pylori*, Tiankuo, Ningbo Xunjian Biotechnology Co., Ltd., Ningbo, China) for *H. pylori* preservation, stored at −20 °C (within 10 days), and processed immediately upon receipt.

Baseline characteristics of the full cohort (*n* = 62) are summarized in Supplementary Table 1, including age, sex, endoscopic and pathological diagnosis, recruitment hospital, and prior treatment history (treatment-naïve vs. prior eradication attempts). The cohort comprised predominantly middle-aged adults with chronic gastritis and peptic ulcer disease as the primary diagnoses, recruited from two hospitals in Zhejiang Province, eastern China.

### 2.2 Bacterial isolation and identification

Biopsies were homogenized and inoculated onto a commercial medium (Gu’s Medium for Rapid Isolation of *Helicobacter pylori*, TianKuo, Ningbo Xunjian Biotechnology Co., Ltd.) to rapidly isolate *H. pylori* (US Patent 18/278,425; European Patent 21927415.6) (Zhang et al., 2023) and then incubated at 37 °C for 2–5 days under microaerophilic conditions using an Anoxomat system (MART Microbiology BV, Drachten, the Netherlands). *Helicobacter pylori* was identified using colony morphology, Gram staining, catalase/oxidase/urease reactions (Gu’s Kit for Rapid Identification of *Helicobacter pylori*, Tiankuo, Ningbo Xunjian Biotechnology Co., Ltd.), and antigen testing (Abon Biopharm, Hangzhou, China), with *H. pylori* ATCC 700824 as the quality control. From each biopsy specimen, 1–10 single colonies were subcultured using the colony suspension dilution method.

Three isolation schemes (a, b, and c) were used to detect mixed infections (Zhang et al., 2023) (Table 1). Scheme a (1–3 single colonies per patient) was used for initial feasibility testing. Scheme b (2–5 colonies) was introduced to explore whether increased sampling improved detection. Scheme c (4 colonies as standard; 4–19 for selected cases) was adopted once we established that 4 isolates balanced detection with practicality, with higher numbers reserved for suspected complex infections. The choice of scheme for each patient depended on enrollment timing and clinical indication. Further details are provided in Supplementary Table 1.

**Table 1:** Isolation scheme distribution and sampling intensity by patient group.

Scheme	*F* group (*n* = 33)		*H* group (*n* = 27)	
	*n* (%)	Isolates/patient	*n* (%)	Isolates/patient
a (1–3)	2 (6.1)	1.50 ± 0.71	18 (66.7)	2.56 ± 0.62
b (2–5)	10 (30.3)	2.10 ± 0.32	1 (3.7)	5
c (≥4)	21 (63.6)	4.00	8 (29.6)	12.25 ± 5.39
Total	33	3.27 ± 0.98	27	5.52 ± 5.35

One quality control case (1 colony) in each group excluded. Scheme a: 1–3 single colonies; b: 2–5 colonies; c: ≥4 colonies (standardized to 4 in F group, 4–19 in selected H group cases). Values are *n* (%) or mean ± SD.

### 2.3 Genomic analysis

Genomic DNA was extracted from single-colony subcultures using a HiPure Bacterial DNA Kit (Guangzhou Magen Biotechnology Co., Ltd., Guangzhou, China), following the manufacturer’s protocol. DNA concentration was measured using a NanoDrop spectrophotometer (Thermo Fisher Scientific, Waltham, MA, USA), and the extracted DNA was stored at −20 °C until use. For the 114 samples, PCR was performed to amplify six flagellar genes (*flaA*, *flaB*, *fliD*, *flgE*, *flgK*, *flgL*) using the primers listed in Supplementary Table 2. The PCR program for flagellar genes was as follows: initial denaturation at 98 °C for 2 min; 30 cycles of denaturation at 98 °C for 10 s, annealing at 55 °C for 30 s, and extension at 72 °C for 20 s; followed by a final extension at 72 °C for 2 min. Seven housekeeping genes (*atpA*, *efp*, *mutY*, *ppa*, *trpC*, *ureI*, and *yphC*) were amplified using the same thermal cycler under the following conditions: initial denaturation at 94 °C for 5 min; 35 cycles of denaturation at 94 °C for 30 s, annealing at the gene-specific temperature for 40 s, and extension at 72 °C for 30–40 s; followed by a final extension at 72 °C for 5 min. The annealing temperatures and extension times were: *atpA* (60 °C, 40 s), *efp* (55 °C, 30 s), *mutY* (55 °C, 30 s), *ppa* (55 °C, 40 s), *trpC* (60 °C, 30 s), *ureI* (60 °C, 30 s), and *yphC* (60 °C, 40 s). PCR products were detected, purified, and Sanger sequenced (Zhang et al., 2023). The obtained sequences were deposited in GenBank under accession numbers PQ035224-PQ035907 (flagellar genes) and PQ606662-PQ607725 (housekeeping genes).

### 2.4 SDR framework

Strain unrelatedness: Strains were considered unrelated when they demonstrated no spatiotemporal correlation, indicating no evidence of epidemiological linkage or mutual exposure.

Genetic profile notation: We developed a notation system to represent groups of isolates sharing identical gene sequences among patients (Tables 2, 3). For example, “ACD (97, 98; 101; 5)” indicates that antral isolates 97 and 98, corpus isolate 101, and duodenal bulb isolate 5 possess identical sequences. Mixed infections were defined as the concurrent presence of multiple distinct genetic profiles.

**Table 2:** Notations of flagellar gene sequence alignments for single-colony isolates from each patient and statistical profiles for each group.

Pt	*flaA*	*flaB*	*fliD*	*flgE*	*flgK*	*flgL*
1	A(1,2)≠C(3, 4)	A(1)≠A(2)≠C(3,4)	A(1)≠A(2)≠C(3,4)	A(1,2)≠C(3,4)	A(1,2)≠C(3,4)	A(1,2)≠C(3,4)
2	ACD(97,98;101;5)	ACD(97,98;101;5)	A(97,98)≠ CD(101;5)	A(97,98)≠ CD(101;5)	ACD(97,98;101;5)	ACD(97,98;101;5)
3	A(6,7)	A(6,7)	A(6,7)	A(6,7)	A(6,7)	A(6,7)
4	D(8,9,10,11)	D(8,9,10,11)	D(8,9,10,11)	D(8,9,10,11)	D(8,9,10,11)	D(8,9,10,11)
5	A(12,13)≠C(14,15)	A(12,13)≠C(14,15)	A(12,13)≠C(14,15)	A(12)≠A(13)≠C(14)≠C(15)	A(12,13)≠C(14,15)	A(12,13)≠C(14,15)
6	A(16,17,18)	A(16,17,18)	A(16,17,18)	A(16,17)≠A(18)	A(16,17,18)	A(16,17)≠A(18)
7	A(19,20,21,22)	A(19,20,21,22)	A(19,20,21,22)	A(19,20,21)≠A(22)	A(19,20,21,22)	A(19,20,21,22)
8	AC(23,24;25,26)	AC(23,24;25,26)	AC(23,24;25,26)	A(23)≠AC(24;25,26)	AC(23,24;25,26)	AC(23,24;25,26)
9	AC(27,28;29,30)	AC(27,28;29,30)	A(28)≠AC(27;29,30)	A(28)≠AC(27;29,30)	AC(27,28;29,30)	AC(27,28;29,30)
10	AC(31,32;33,34)	AC(31,32;33,34)	A(31,32)≠C(33,34)	AC(31,32;33,34)	AC(31,32;33,34)	A(31,32)≠C(33,34)
11	C(35,36,37,38)	C(35,36,37,38)	C(35,37,38)≠C(36)	C(35,36,37,38)	C(35,36,37,38)	C(35,36,37,38)
12	A(39,40)	A(39,40)	A(39,40)	A(39,40)	A(39,40)	A(39,40)
13	A(41,42,43,44)	A(41,42,43,44)	A(41,42,43,44)	A(41,42,43,44)	A(41,42,43,44)	A(41,42,43,44)
14	AC(102,103;45,46)	AC(102,103;45,46)	AC(102,103;45,46)	AC(102,103;45,46)	AC(102,103;45,46)	AC(102,103;45,46)
15	C(47)≠D(104)	CD(47;104)	CD(47;104)	CD(47;104)	CD(47;104)	C(47)≠D(104)
16	C(48)≠C(49)	C(48,49)	C(48,49)	C(48,49)	C(48,49)	C(48,49)
17	ACD(50,51;52;57)	ACD(50,51;52;57)	ACD(50,51;52;57)	ACD(50,51;52;57)	ACD(50,51;52;57)	A(51)≠ACD(50;52;57)
18	ACD(53,54;55;56)	ACD(53,54;55;56)	ACD(53,54;55;56)	ACD(53,54;55;56)	ACD(53,54;55;56)	ACD(53,54;55;56)
19	ACD(58,59;60;61)	ACD(58,59;60;61)	ACD(58,59;60;61)	ACD(58,59;60;61)	ACD(58,59;60;61)	ACD(58,59;60;61)
20	ACD(67,68;62;69)	ACD(67,68;62;69)	ACD(67,68;62;69)	ACD(67,68;62;69)	ACD(67,68;62;69)	ACD(67,68;62;69)
21	A(63)≠AC(64;65,66)	AC(63,64;65,66)	AC(63,64;65,66)	AC(63,64;65,66)	AC(63,64;65,66)	AC(63,64;65,66)
22	AC(70,71;72,96)	AC(70,71;72,96)	AC(70,71;72)≠C(96)	AC(70,71;72)≠C(96)	AC(70,71;72,96)	AC(70,71;72)≠C(96)
23	AC(73,74;75,76)	AC(73,74;75,76)	AC(73,74;75,76)	A(73,74)≠C(75)≠C(76)	AC(73,74;75,76)	AC(73,74;75,76)
24	AC(77,78;79,80)	AC(77,78;79,80)	AC(77,78;79,80)	AC(77,78;79,80)	AC(77,78;79,80)	AC(77,78;79,80)
25	CD(81,82;83,84)	CD(81,82;83,84)	CD(81,82;83,84)	CD(81,82;83,84)	CD(81,82;83)≠D(84)	CD(81,82;83,84)
26	A(85)≠A(86)	A(85,86)	A(85,86)	A(85,86)	A(85,86)	A(85,86)
27	A(87,88)	A(87,88)	A(87,88)	A(87,88)	A(87,88)	A(87,88)
28	A(89,90,91,92)	A(89,90,91,92)	A(89,90,91,92)	A(89,90,91,92)	A(89,90,91,92)	A(89,90,91,92)
29	D(93,94)	D(93,94)	D(93,94)	D(93,94)	D(93,94)	D(93,94)
30	AD(99,100;105,106)	AD(99,100;105,106)	AD(99,100;105,106)	AD(99,100;105,106)	AD(99,100;105,106)	AD(99,100;105,106)
31	A(107,108)	A(107,108)	A(107,108)	A(107,108)	A(107,108)	A(107,108)
32	A(109,110)	A(109,110)	A(109,110)	A(109,110)	A(109,110)	A(109,110)
33	D(111,112)	D(111,112)	D(111,112)	D(111,112)	D(111,112)	D(111,112)
34	C(95)	C(95)	C(95)	C(95)	C(95)	C(95)
*n*	40	37	42	46	37	41
C	780	666	861	1035	666	820
NI	1	0	0	0	0	0
ACQ	6	4	9	16	3	7
CO	773	662	852	1019	663	813
Max	4.25%	3.50%	2.88%	3.03%	4.46%	3.70%
Min	0.98%	0.45%	0.05%	0.28%	1.16%	1.25%
p*	0.000	0.025	0.001	0.000	0.000	0.042
Med	2.22%	2.14%	2.13%	2.14%	2.20%	2.33%
Mo	1.83%	2.20%	2.23%	2.19%	1.98%	2.33%
Sk	68.56%	3.77%	−52.56%	−15.32%	88.76%	16.18%
*p*1	1.31%	1.04%	1.49%	1.40%	1.38%	1.57%
*p*99	3.66%	3.11%	2.73%	2.89%	3.97%.	3.18%
RR*	1.31–3.66%	1.04–3.11%	1.49–2.73%	1.40–2.89%	1.38–3.97%	1.57–3.18%

*n*, number of profiles per group; C, total count of sequence difference ratio (SDR) values per group; NI, number of instances with SDR = 0 between strains from different patients; ACQ, total combination quantity for diverse profiles in each patient with mixed infection, calculated as the sum of C(*r*, 2) = *r*(*r* − 1)/2, where *r* is the number of profiles per mixed-infected patient; CO, count of observations per group profile, calculated as C–NI–ACQ; p*, *p*-value from Kolmogorov–Smirnov normality test; Med, median; Mo, mode; Sk, skewness; RR, reference ranges of SDR values for all profiles within each group (Equation 1).

**Table 3:** Notations of housekeeping gene sequence alignments for single-colony isolates and group-specific statistical profiles.

Pt	*atpA*	*efp*	*mutY*	*ppa*	*trpC*	*ureI*	*yphC*
h1	A(H13)≠CD(H21;H10)	AD(H13;H10)≠ C(H21)	ACD(H13;H21;H10)	ACD(H13;H21;H10)	AD(H13;H10)≠ C(H21)	ACD(H13;H21;H10)	ACD(H13;H21;H10)
h2	ACD(H11;H22;H12)	ACD(H11;H22;H12)	ACD(H11;H22;H12)	ACD(H11;H22;H12)	ACD(H11;H22;H12)	ACD(H11;H22;H12)	ACD(H11;H22;H12)
h3	CD(H14;H15)	CD(H14;H15)	CD(H14;H15)	CD(H14;H15)	CD(H14;H15)	CD(H14;H15)	CD(H14;H15)
h4	AD(H20;H19)≠C(18)	ACD(H20;H18;H19)	ACD(H20;H18;H19)	ACD(H20;H18;H19)	ACD(H20;H18;H19)	ACD(H20;H18;H19)	ACD(H20;H18;H19)
h5	A(H23)≠C(H42)	A(H23)≠C(H42)	A(H23)≠C(H42)	A(H23)≠C(H42)	A(H23)≠C(H42)	A(H23)≠C(H42)	A(H23)≠C(H42)
h6	CD(H24;H44)	CD(H24;H44)	CD(H24;H44)	CD(H24;H44)	CD(H24;H44)	CD(H24;H44)	CD(H24;H44)
h7	ACD(H54;H25;H26)	ACD(H54;H25;H26)	ACD(H54;H25;H26)	ACD(H54;H25;H26)	ACD(H54;H25;H26)	ACD(H54;H25;H26)	ACD(H54;H25;H26)
h8	C(H27,H28,H29,H30,H31,H32,H33)	C(H27,H28,H29,H30,H31,H32,H33)	C(H27,H28,H29,H30,H31,H32,H33)	C(H27,H28,H29,H30,H31,H32,H33)	C(H27,H28,H29,H30,H31,H32,H33)	C(H27,H28,H29,H30,H31,H32,H33)	C(H27,H28,H29,H30,H31,H32,H33)
h9	D(H34,H35,H36,H37,H38,H39,H40,H41)	D(H34,H35,H36,H37,H38,H39,H40,H41)	D(H34,H35,H36,H37,H38,H39,H40,H41)	D(H34,H35,H36,H37,H38,H39,H40,H41)	D(H34,H35,H36,H37,H38,H39,H40,H41)	D(H34,H35,H36,H37,H38,H39,H40,H41)	D(H34,H35,H36,H37,H38,H39,H40,H41)
h10	ACD(H45;H46,H48;H47)	ACD(H45;H46,H48;H47)	ACD(H45;H46,H48;H47)	ACD(H45;H46,H48;H47)	ACD(H45;H46,H48;H47)	ACD(H45;H46,H48;H47)	ACD(H45;H46,H48;H47)
h11	A(H17)≠C(H9)	AC(H17;H9)	AC(H17;H9)	AC(H17;H9)	AC(H17;H9)	AC(H17;H9)	AC(H17;H9)
h12	ACD(H50;H94;H51)	ACD(H50;H94;H51)	ACD(H50;H94;H51)	ACD(H50;H94;H51)	ACD(H50;H94;H51)	ACD(H50;H94;H51)	ACD(H50;H94;H51)
h13	ACD(H86;H87;H52)	ACD(H86;H87;H52)	ACD(H86;H87;H52)	ACD(H86;H87;H52)	ACD(H86;H87;H52)	ACD(H86;H87;H52)	A(H86) ≠ CD(H87;H52)
h14	ACD(H92;H53;H93)	ACD(H92;H53;H93)	AC(H92;H53)≠ D(H93)	ACD(H92;H53;H93)	ACD(H92;H53;H93)	ACD(H92;H53;H93)	ACD(H92;H53;H93)
h15	AD(H55,H56,H59,H60,H61,H62,H97;H63,H64)≠AD(H57,H58;H65,H66,H68,H69,H70) ≠D(H67)	AD(H55,H56,H57,H58,H59,H60,H61,H62,H97;H63,H64,H65,H66,H67,H68,H69,H70)	AD(H55,H56,H57,H58,H59,H60,H61,H62,H97;H63,H64,H65,H66,H67,H68,H69,H70)	AD(H55,H56,H57,H58,H59,H60,H61,H62,H97;H63,H64,H65,H66,H67,H68,H69,H70)	AD(H55,H56,H57,H58,H59,H60,H61,H62,H97;H63,H64,H65,H66,H67,H68,H69,H70)	AD(H55,H56,H57,H58,H59,H60,H61,H62,H97;H63,H64,H65,H66,H67,H68,H69,H70)	AD(H55,H56,H57,H58,H59,H60,H61,H62,H97;H63,H64,H65,H66,H67,H68,H69,H70)
h16	A(H71,H72,H73,H74,H76,H77,H78)≠A(H75)≠C(H79,H80,H81,H82,H83,H84,H85)	A(H71,H72,H73,H74,H75,H76,H77,H78)≠C(H79,H80,H81,H82,H83,H84,H85)	A(H71,H72,H73,H74,H75,H76,H77,H78)≠C(H79,H80,H81,H82,H83,H84,H85)	A(H71,H72,H73,H74,H75,H76,H77,H78)≠C(H79,H80,H81,H82,H83,H84,H85)	A(H71,H72,H73,H74,H75,H76,H77,H78)≠C(H79,H80,H81,H82,H83,H84,H85)	A(H71,H72,H73,H74,H75,H76,H77,H78)≠C(H79,H80,H81,H82,H83,H84,H85)	A(H71,H72,H73,H74,H75,H76,H77,H78)≠C(H79,H80,H81,H82,H83,H84,H85)
h17	ACD(H88;H89;H95)	ACD(H88;H89;H95)	ACD(H88;H89;H95)	ACD(H88;H89;H95)	ACD(H88;H89;H95)	ACD(H88;H89;H95)	ACD(H88;H89;H95)
h18	ACD(H101;H90;H91)	ACD(H101;H90;H91)	ACD(H101;H90;H91)	ACD(H101;H90;H91)	ACD(H101;H90;H91)	ACD(H101;H90;H91)	ACD(H101;H90;H91)
h19	ACD(H96;H98;H99)	ACD(H96;H98;H99)	ACD(H96;H98;H99)	ACD(H96;H98;H99)	ACD(H96;H98;H99)	ACD(H96;H98;H99)	ACD(H96;H98;H99)
h20	ACD(H100;H102;H103)	ACD(H100;H102;H103)	ACD(H100;H102;H103)	ACD(H100;H102;H103)	ACD(H100;H102;H103)	ACD(H100;H102;H103)	ACD(H100;H102;H103)
h21	AC(H104;H105)	AC(H104;H105)	AC(H104;H105)	AC(H104;H105)	AC(H104;H105)	AC(H104;H105)	AC(H104;H105)
h22	CD(H108;H106)	CD(H108;H106)	CD(H108;H106)	CD(H108;H106)	CD(H108;H106)	CD(H108;H106)	CD(H108;H106)
h23	AC(H109;H107)	AC(H109;H107)	AC(H109;H107)	AC(H109;H107)	AC(H109;H107)	AC(H109;H107)	AC(H109;H107)
h24	A(H113,H114,H115,H116,H121,H122,H123,H124)≠C(H117,H118,H119,H120)	A(H113,H114,H115,H116,H121,H122,H123,H124)≠C(H117,H118,H119,H120)	A(H113,H114,H115,H116,H121,H122,H123,H124) ≠C(H117,H118,H119,H120)	A(H113,H114,H115,H116,H121,H122,H123,H124)≠C(H117,H118,H119,H120)	A(H113,H114,H115,H116,H121,H122,H123,H124)≠C(H117,H118,H119,H120)	A(H113,H114,H115,H116,H121,H122,H123,H124)≠C(H117,H118,H119,H120)	A(H113,H114,H115,H116,H121,H122,H123,H124)≠C(H117,H118,H119,H120)
h25	AD(H125,H126,H127,H128,H129,H130,H131,H132;H133,H134,H135,H136,H137,H138)≠C(H139)≠C(H140)	AD(H125,H126,H127,H128,H129,H130,H131,H132;H133,H134,H135,H136,H137,H138)≠C(H139)≠C(H140)	AD(H125,H126,H127,H128,H129,H130,H131,H132;H133,H134,H135,H136,H137,H138)≠C(H139)≠C(H140)	AD(H125,H126,H127,H128,H129,H130,H131,H132;H133,H134,H135,H136,H137,H138)≠C(H139)≠C(H140)	AD(H125,H126,H127,H128,H129,H130,H131,H132;H133,H134,H135,H136,H137,H138)≠C(H139)≠C(H140)	AD(H125,H126,H127,H128,H129,H130,H131,H132;H133,H134,H135,H136,H137,H138)≠C(H139)≠C(H140)	AD(H125,H126,H127,H128,H129,H130,H131,H132;H133,H134,H135,H136,H137,H138)≠C(H139)≠C(H140)
h26	A(H141,H142,H143,H144,H145)	A(H141,H142,H143,H144,H145)	A(H141,H142,H143,H144,H145)	A(H141,H142,H143,H144,H145)	A(H141,H142,H143,H144,H145)	A(H141,H142,H143,H144,H145)	A(H141,H142,H143,H144,H145)
h27	A(H156)≠A(H160)≠AC(H146,H147,H148,H149,H155,H157,H158,H159;H150,H151,H152,H153,H154,H161,H162,H163, H164)	A(H156)≠AC(H146,H147,H148,H149,H155,H157,H158,H159,H160;H151,H162,H163)≠C(H150,H152,H153,H154,H161,H164)	AC(H146,H147,H148,H149,H155,H156,H157,H158,H159,H160;H150,H151,H152,H153,H154,H161,H162,H163,H164)	A(H156)≠AC(H146,H147,H148,H149,H155,H157,H158,H159,H160;H150,H151,H152,H153,H154,H161,H162,H163,H164)	A(H156)≠AC(H146,H147,H148,H149,H155,H157,H158,H159,H160;H150,H151,H152,H153,H154,H161,H162,H163,H164)	A(H147,H156)≠AC(H146,H148,H149,H155,H157,H158,H159,H160;H150,H151,H152,H153,H154,H161,H162,H163,H164)	AC(H146,H147,H148,H149,H155,H156,H157,H158,H159,H160;H150,H151,H152,H153,H154,H161,H162,H163,H164)
h28	D(H49)	D(H49)	D(H49)	D(H49)	D(H49)	D(H49)	D(H49)
n	41	36	34	34	35	34	34
C	820	630	561	561	595	561	561
NI	17	2	2	4	3	2	4
ACQ	17	10	7	7	8	7	7
CO	786	618	552	550	584	552	550
Max	3.83%	4.69%	5.95%	5.43%	29.87%	4.15%	5.50%
Min	0.16%	0.25%	0.24%	0.26%	0.88%	0.17%	0.39%
P*	0.000	0.000	0.000	0.000	0.000	0.000	0.000
Med	1.59%	2.47%	3.57%	1.29%	2.88%	1.55%	2.16%
Mo	1.28%	2.22%	3.33%	1.03%	2.65%	1.04%	1.96%
Sk	14.22%	17.11%	−13.08%	177.03%	330.57%	77.70%	76.87%
p5	0.80%	1.23%	2.14%	0.52%	1.77%	0.69%	0.98%
p95	2.55%	3.70%	4.76%	3.62%	24.30%	2.84%	4.52%
RR	0.80–2.55%	1.23–3.70%	2.14–4.76%	0.52–3.62%	1.77–24.30%	0.69–2.84%	0.98–4.50%

*n*, number of profiles per group; C, total count of sequence difference ratio (SDR) values per group; NI, number of instances with SDR = 0 between strains from different patients; ACQ, total combination quantity for diverse profiles in each patient with mixed infection, calculated as the sum of C(*r*, 2) = *r*(*r* − 1)/2, where *r* is the number of profiles per mixed-infected patient; CO, count of observations per group profile, calculated as C–NI–ACQ; p*, *p*-value from Kolmogorov–Smirnov normality test; Med, median; Mo, mode; Sk, skewness; RR, reference ranges of SDR values for all profiles within each group (Equation 1).

#### 2.4.1 SDR calculation

SDR was calculated as follows:

SDR=(NDS/LATGS)×100% (1)

where NDS is the number of single-nucleotide polymorphisms, and LATGS is the total alignment length. Gene-specific alignment lengths were as follows: for flagellar genes, *flaA* (1,530 bp), *flaB* (1,542 bp), *fliD* (2,016 bp), *flgE* (2,148 bp), *flgK* (1,815 bp), and *flgL* (2,487 bp); for housekeeping genes, *atpA* (627 bp), *efp* (405 bp), *mutY* (420 bp), *ppa* (387 bp), *trpC* (452 bp), *ureI* (579 bp), *yphC* (509 bp). SDR = 0 indicates identical sequences. Unrelated strains typically yield non-zero SDRs, although rare zero-SDR cases reflect local sequence identity in otherwise divergent strains.

SDR values were computed using Microsoft Excel 2007 (Microsoft, Redmond, WA, USA). For each gene, pairwise alignments among “*n*” profiles generated C = *n*(*n*–1)/2 comparisons. Alignments with zero SDR (NI) and acquired sequences (ACQ) were excluded, yielding a final count of CO = C–NI–ACQ (Table 2). The complete pairwise sequence alignment data are presented in Tables 2, 3.

#### 2.4.2 Establishment of SDR reference ranges

The SDR distributions were assessed using SPSS (version 27.0; SPSS Inc., Chicago, IL, USA). For normally distributed data, reference ranges were defined as mean ± 2 standard deviations. For non-normally distributed data, percentile-based methods were applied: 1st–99th percentiles for flagellar genes and 5th–95th percentiles for housekeeping genes.

### 2.5 Clustering of multigene alteration patterns

We established a dataset documenting the gene alteration proportions across isolates for six (flagellar) and seven (housekeeping) gene panels to examine the distribution and frequency of genetic alterations.

### 2.6 Statistical analysis

Analyses were performed using SPSS software (version 27.0; SPSS Inc.). We compared the gene-specific mixed infection proportions and evaluated single- and dual-gene alteration patterns. Higher-order combinatorial alterations (three to seven genes) were investigated using a mixed-infection mechanism analysis.

$$\text{Proportion}=(\frac{\begin{array}{c} \text{number of alteration events}\;\\ \text{for specific patterns} \end{array}}{\text{total alteration events}})\times 100\%$$ (2)

Hierarchical clustering was performed using between-group linkages with Euclidean distances, allowing the identification of distinct strain-specific alteration profiles.

### 2.7 Revelation of mixed infection mechanisms

We implemented a hierarchical classification system based on SDR reference ranges derived from pairwise sequence alignments of both flagellar and housekeeping gene profiles.

#### 2.7.1 Gene-level variation interpretation

For each gene, pairwise SDR values were evaluated against the established lower reference limit.

Gene mutation: Pairwise SDR values for the gene fell below the lower reference limit.

Gene recombination: Exactly one alignment (maximum two) showed SDR at or above the lower reference limit, while all remaining alignments had SDR values of 0 or were below the lower reference limit.

Genetic unrelatedness: Most or all pairwise SDR values reached or exceeded the lower reference limit, indicating extensive sequence divergence across comparisons.

Heterologous recombination (interspecies horizontal gene transfer) is a distinct subclass of gene recombination. For each gene, the upper-to-lower limit ratio (ULR) was calculated as the upper bound divided by the lower bound of the SDR range. A markedly elevated ULR (ULR > 10) served as the initial screen. Confirmatory identification required that the focal gene SDR range, calculated from pairwise comparisons between the focal strain and all other strains, substantially exceed both the upper bound of the local reference range and the SDR reference range established from the global reference strain (external reference strain, e.g., *H. pylori* ATCC 700824).

#### 2.7.2 Strain relationship classification

Related strains: Encompass strains failing to meet unrelated strain criteria, categorized into gene-locus mutations, gene recombination, or combined mutation–recombination profiles.

Unrelated strains: Classified when all six flagellar or seven housekeeping genes demonstrated genetic unrelatedness, or when no more than two genes exhibited patterns potentially indicative of convergent mutations.

Superinfection: Identified by the concurrent presence of two or more unrelated strains within a single individual during the same infection period.

The hierarchical classification workflow is summarized in Figure 1.

**Figure 1:**
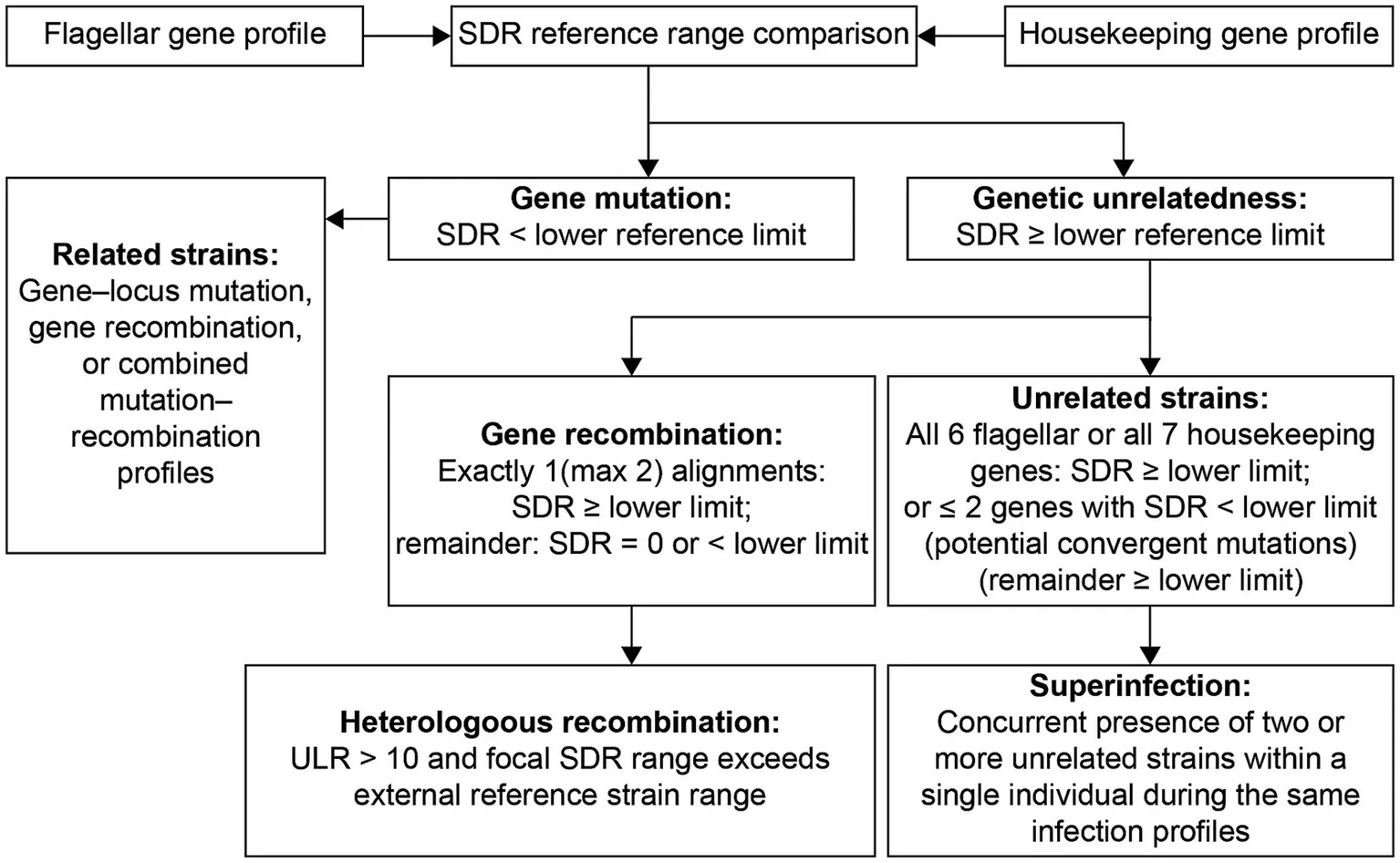
Hierarchical classification for mixed infection mechanism. SDR-based decision hierarchy for classifying *H. pylori* mixed-infection mechanisms. Pairwise alignments of flagellar and housekeeping genes are evaluated against the sequence difference ratio (SDR) reference-range framework. Gene-level classifications (mutation, recombination, genetic unrelatedness, and heterologous recombination) are determined by SDR values relative to the lower reference limit, alignment count, and the upper-to-lower-limit ratio (ULR). Strain-level categories (related, unrelated, superinfection) are assigned by aggregating gene-level outcomes. For full classification criteria, see Section 2.7.

### 2.8 Geographical divergence assessment

Geographical divergence of strain origin was inferred from SDR values across multiple gene loci. Divergence was considered present if the SDR values of an isolate exceeded the predefined regional reference ranges for at least three of the six flagellar genes (*flaA*, *flaB*, *fliD*, *flgE*, *flgK*, and *flgL*) or for at least three of the seven housekeeping genes (*atpA*, *efp*, *mutY*, *ppa*, *trpC*, *ureI*, and *yphC*). To implement this assessment, gene sequences from reference strains ATCC 700824 and H54 were aligned with those of clinical isolates, and SDR values were calculated and compared against gene-specific reference ranges.

### 2.9 Phylogenetic validation of the SDR framework

We performed independent phylogenetic validations across all 13 genetic loci. Thirteen gene-specific trees were constructed using the neighbor-joining method in MEGA 11 (Zhang et al., 2023). To visualize these relationships, strains from the same patient were colored identically across all trees. The topological patterns of each tree were systematically compared with the corresponding gene-specific SDR values. The strong congruence between phylogenetic clustering, particularly the co-clustering of same-patient strains, and SDR-based classifications validated the accuracy of the framework for distinguishing related strains, recombination events, and divergent lineages. The observed discrepancies were analyzed to refine the SDR thresholds. This direct comparison confirms that the SDR framework provides enhanced resolution over traditional phylogenetics for differentiating recent intrahost microevolution from deeper phylogenetic divergence, which is indicative of superinfection.

### 2.10 Statistical power analysis

Post-hoc power calculations were performed using G*Power 3.1.9.7 (Heinrich-Heine-Universität Düsseldorf, Germany). The primary comparison, superinfection rates between flagellar and housekeeping gene categories, was analyzed using a two-proportion *z*-test (*α* = 0.05, two-tailed). The dominant mutation pattern in flagellar genes was evaluated using a one-proportion *z*-test against a 50% null hypothesis. Full parameters and results are reported in Supplementary Table 3.

### 2.11 BLASTn and phylogenetic analysis of *trpC*

The *trpC* sequence of strain H42 was queried against the NCBI nucleotide database using BLASTn to retrieve closely related sequences. These retrieved sequences were combined with the *trpC* sequences generated in this study and the sequence of the reference strain ATCC 700824 (all deposited in NCBI) for phylogenetic reconstruction. A maximum likelihood tree was constructed using MEGA 6.0 under the Kimura 2-parameter model, and node support was assessed using 1,000 bootstrap replicates.

## 3 Results

### 3.1 Patient characteristics and high recovery rates under delayed processing conditions

One patient in each group had only one colony recovered and was treated as a quality control, leaving 33 and 27 patients for analysis. Age, sex, and treatment history were similar between groups (all *p* > 0.05). However, disease severity differed between the groups: the F group had higher proportions of patients with ulcer disease (67.6% vs. 28.6%) and combined lesions (8.8% vs. 0%), whereas the H group predominantly comprised patients with gastritis (71.4% vs. 23.5%) (*p* < 0.001). This difference likely reflects sequential enrollment, as F group patients were recruited earlier, when more severe cases were referred (Table 4). Full details are provided in Supplementary Table 1.

**Table 4:** Primary growth of *H. pylori* in biopsy specimens from the F group.

Pt #	Antrum		Corpus		Duodenal bulb	
	DFAC	NC	DFAC (d)	NC	DFAC (d)	NC
1	2d	++++	2d	++++	2d	++++
2	3d	++	3d	++++	3d	++++
3	2d	++++	3d	++++	3d	++++
4	5d	0	3d	+	3d	+
5	3d	++++	3d	++	3d	++++
6	3d	+++	3d	++	N	N
7	3d	+++	3d	++	N	N
8	2d	+	3d	++++	N	N
9	2d	++++	3d	++++	N	N
10	3d	++++	3d	++++	N	N
11	3d	++++	2d	++++	3d	++++
12	3d	++++	2d	++++	3d	++++
13	3d	++++	2d	++++	3d	+
14	3d	++++	2d	++++	3d	++++
15	3d	+++	3d	+++	3d	6
16	2d	++++	3d	++++	3d	+
17	3d	++++	2d	++++	3d	++++
18	3d	++++	3d	++++	3d	++++
19	3d	++++	3d	++	3d	++++
20	2d	++++	2d	++++	3d	++++
21	3d	++	3d	++++	3d	++++
22	3d	++++	3d	++++	3d	++++
23	3d	++++	3d	++++	N	N
24	3d	++++	3d	++++	2d	++++
25	5d	0	3d	++++	3d	++++
26	3d	+++	2d	++	3d	++++
27	3d	++++	2d	++++	3d	+++
28	3d	++++	2d	+++	3d	++++
29	3d	++++	N	N	3d	++++
30	3d	+++	3d	++++	3d	++++
31	3d	++++	3d	++++	3d	+++
32	3d	++++	3d	+	3d	++
33	3d	++++	3d	++	3d	++++
34	3d	++	3d	++++	3d	++++

Delay period ranged from 3 to 6 h. DFAC, days to first appearance of colonies; NC, number of colonies; CFU, colony-forming unit. All Gu’s plates were monitored for semiquantitative *H. pylori* growth: +, 1–10^2^ CFU (scant growth); ++, 10^2^–10^3^ CFU (sparse growth); +++, semi-confluent growth; ++++, confluent growth. N, no data; #, patients at the Affiliated Hospital of Ningbo University School of Medicine; Pt, patient.

The two groups also differed in how the schemes were applied. The F group mostly used scheme c (21/33, 63.6%), with a fixed 4 colonies per patient. The H group mostly used scheme a (18/27, 66.7%), with 1–3 colonies, and selective scheme c for complex cases (8/27, 29.6%). This shift from fixed to flexible sampling reflects how the study evolved. Mean intensity was 3.27 ± 0.98 in the F group and 5.52 ± 5.35 in the H group; the wider spread in the H group comes from targeted high-intensity sampling (Table 1). Per-patient details are in Supplementary Table 1.

### 3.2 Quality control

*Helicobacter pylori* reference strain ATCC 700824 (J99) was used as a positive control throughout all experimental procedures. Identification was performed using morphological assessment, biochemical characterization, and antigen detection. Sanger sequencing of all 13 gene loci generated sequences identical to those of the reference genome ATCC 700824 (J99) (GenBank accession No. AE001439), which confirmed the specificity, reliability, and accuracy of the experimental workflow.

### 3.3 Determination of SDR reference ranges

Analysis of 6 flagellar genes from 112 isolates (33 patients) revealed mixed infections in 51.5% (17/33) of patients. Sequence alignment of isolates 113 and 114 with the reference strain ATCC 700824 confirmed methodological reliability. All flagellar gene datasets were non-normally distributed (Kolmogorov–Smirnov test, *p* < 0.05). Reference ranges (1st–99th percentiles) were established: *flaA* (1.31–3.66%), *flaB* (1.04–3.11%), *fliD* (1.49–2.73%), *flgE* (1.40–2.89%), *flgK* (1.38–3.97%), and *flgL* (1.57–3.18%) (Table 2). The corresponding ULR values (1.83–2.99) were all below the predefined threshold of 10, consistent with the absence of heterologous recombination signals among flagellar genes.

Analysis of 7 housekeeping genes in 27 patients revealed mixed infections (Table 3). All gene datasets were non-normally distributed (*p* < 0.05). The reference ranges (5th–95th percentiles) were as follows: *atpA* (0.80–2.55%), *efp* (1.23–3.70%), *mutY* (2.14–4.76%), *ppa* (0.52–3.62%), *trpC* (1.77–24.30%), *ureI* (0.69–2.84%), and *yphC* (0.98–4.50%). The ULR of the SDR reference ranges were *atpA* (3.19), *efp* (3.01), *mutY* (2.22), *ppa* (6.96), *trpC* (13.73), *ureI* (4.12), and *yphC* (4.59). While the ULR values for six genes ranged from 2.22 to 6.96, *trpC* exhibited a markedly elevated ratio, approximately 2- to 6-fold higher than those of any other locus, indicating an exceptionally broad variation interval and a substantially expanded upper limit (Table 3).

### 3.4 Flagellar gene analysis

Analysis of 6 flagellar genes from 112 isolates detected mixed infections in 51.5% (17/33) of the patients. Hierarchical clustering of gene alteration patterns identified two distinct groups: Cluster 1 (single−/double−/triple-gene events) and Cluster 2 [six-gene concurrent alterations, 35.3% (12/34)] (Figure 2 and Supplementary Table 4; Equation 2). Among the 17 patients with mixed infections, SDR classification revealed 3 evolutionary mechanisms: mutation in 82.4% (14/17), recombination in 5.9% (1/17), and superinfection-associated mechanisms in 11.8% (2/17), the latter including convergent mutations in the *fliD* locus (Table 5).

**Figure 2:**
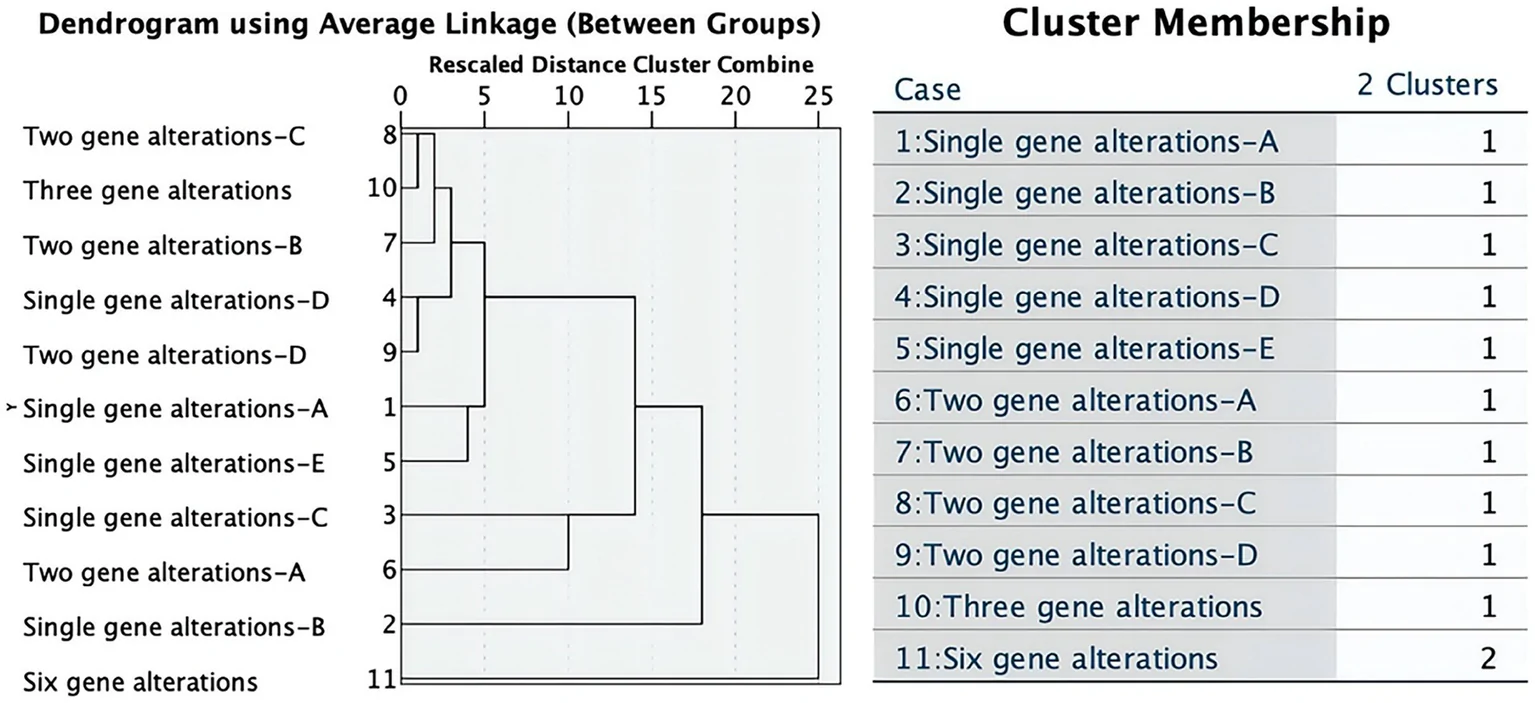
Clustering of flagellar gene alterations in *H. pylori*.

**Table 5:** Mechanistic analysis of *H. pylori* mixed infections in the F group using the SDR method.

Pt	Gene	CDS	SDR	CGEP	EM
1	*flaA*	A(1,2) vs. C(3,4)=37	2.42%	U	A(1) and A(2): Related strains with *flaB* and *fliD* mutations. A(1) and C(3,4): Unrelated strains exhibiting genetic unrelatedness across all six flagellar gene loci. A(2) and C(3,4): Unrelated strains exhibiting convergent mutations in the *fliD* locus. Pt 1: Superinfection involving both unrelated and related strains, exhibiting three patterns (SMCM).
	*flaB*	A(1) vs. A(2)=5 A(1) vs. C(3,4)=17 A(2) vs. C(3,4)=22	0.32% 1.10% 1.43%	M U U	
	*fliD*	A(1) vs A(2)=29 A(1) vs. C(3,4)=30 A(2) vs. C(3,4)=3	1.44% 1.49%, 0.15%	M U CM	
	*flgE*	A(1,2) vs. C(3,4)=42	1.96%	U	
	*flgK*	A(1,2) vs. C(3,4)=31	1.71%	U	
	*flgL*	A(1,2) vs. C(3,4)=58	2.33%	U	
2	*flaA*	ACD(97,98;101;5)	0		A(97,98) and CD(101;5): Related strains with *fliD* and *flgE* locus-specific mutations. Pt 2: Related strains with single mutations in two flagellin loci (M).
	*flaB*	ACD(97,98;101;5)	0		
	*fliD*	A(97,98) vs. CD(101;5)=1	0.050%	M	
	*flgE*	A(97,98) vs. CD(101;5)=1	0.047%	M	
	*flgK*	ACD(97,98;101;5)	0		
	*flgL*	ACD(97,98;101;5)	0		
5	*flaA*	A(12,13) vs. C(14,15)=42	2.75%	U	A(12) and A(13): Related strains demonstrating *flgE* recombination. C(14) and C(15): Related strains with *flgE* mutations. A(12) and C(14): Unrelated strains. A(12) and C(15): Unrelated strains. A(13) and C(14): Unrelated strains. A(13) and C(15): Unrelated strains.Pt 5: Superinfection involving both unrelated and related strains, exhibiting two patterns (SMR).
	*flaB*	A(12,13) vs. C(14,15)=32	2.08%	U	
	*fliD*	A(12,13) vs. C(14,15)=37	1.84%	U	
	*flgE*	A(12) vs. A(13)=40	1.86%	R	
		C(14) vs. C(15)=13	0.61%	M	
		A(13) vs. C(14)=46	2.14%	U	
		A(12) vs. C(14)=37	1.72%	U	
		A(12) vs. C(15)=34	1.58%	U	
		A(13) vs. C(15)=52	2.42%	U	
	*flgK*	A(12,13) vs. C(14,15)=51	2.81%	U	
	*flgL*	A(12,13) vs. C(14,15)=51	2.05%	U	
6	*flaA*	A(16,17,18)	0		A(16,17) and A(18): Related strains with *flgE* loci and *flgL* multilocus mutations. Pt 6: Related strains with dual flagellin locus mutations (M).
	*flaB*	A(16,17,18)	0		
	*fliD*	A(16,17,18)	0		
	*flgE*	A(16,17) vs. A(18)=9	0.42%	M	
	*flgK*	A(16,17,18)	0		
	*flgL*	A(16,17) vs. A(18)=1	0.040%	M	
7	*flaA*	A(19,20,21,22)	0		A(19,20,21) and A(22): Related strains with *flgE* locus mutations. Pt 7: Related strains with flagellin locus mutations (M).
	*flaB*	A(19,20,21,22)	0		
	*fliD*	A(19,20,21,22)	0		
	*flgE*	A(19,20,21) vs A(22)=12	0.56%	M	
	*flgK*	A(19,20,21,22)	0		
	*flgL*	A(19,20,21,22)	0		
8	*flaA*	AC(23,24;25,26)	0		A(23) and AC(24;25,26): Related strains with *flgE* locus mutations. Pt 8: Related strains with flagellin locus mutations (M).
	*flaB*	AC(23,24;25,26)	0		
	*fliD*	AC(23,24;25,26)	0		
	*flgE*	A(23) vs. AC(24;25,26)=9	0.42%	M	
	*flgK*	AC(23,24;25,26)	0		
	*flgL*	AC(23,24;25,26)	0		
9	*flaA*	AC(27,28;29,30)	0		A(28) and AC(27;29,30): Related strains with *fliD* loci and *flgE* mutations. Pt 9: Related strains with dual flagellin locus mutations (M).
	*flaB*	AC(27,28;29,30)	0		
	*fliD*	A(28) vs. AC(27;29,30)=15	0.74%	M	
	*flgE*	A(28) vs. AC(27;29,30)=1	0.047%	M	
	*flgK*	AC(27,28;29,30)	0		
	*flgL*	AC(27,28;29,30)	0		
10	*flaA*	AC(31,32;33,34)	0		A(31,32) and C(33,34): Related strains with single mutations in *fliD* and *flgL* loci. Pt 10: Related strains with dual flagellin locus mutations (M).
	*flaB*	AC(31,32;33,34)	0		
	*fliD*	A(31,32) vs. C(33,34)=1	0.050%	M	
	*flgE*	AC(31,32;33,34)	0		
	*flgK*	AC(31,32;33,34)	0		
	*flgL*	A(31,32) vs. C(33,34)=1	0.040%	M	
11	*flaA*	C(35,36,37,38)	0		C(35,37,38) and C(36): Related strains with single *fliD* locus mutation. Pt 11: Related strains with flagellar locus mutation (M).
	*flaB*	C(35,36,37,38)	0		
	*fliD*	C(35,37,38) vs. C(36)=1	0.050%	M	
	*flgE*	C(35,36,37,38)	0		
	*flgK*	C(35,36,37,38)	0		
	*flgL*	C(35,36,37,38)	0		
15	*flaA*	C(47) vs. D(104)=1	0.065%	MUT	C(47) and (104): Related strains with *flaA* and *flgL* locus mutations. Pt 15: Related strains with dual flagellin locus mutations (M).
	*flaB*	CD(47;104)	0		
	*fliD*	CD(47;104)	0		
	*flgE*	CD(47;104)	0		
	*flgK*	CD(47;104)	0		
	*flgL*	C(47) vs. D(104)=20	0.80%	M	
16	*flaA*	C(48) vs. C(49)=1	0.065%	M	C(48) and C(49): Related strains with *flaA* locus mutation. Pt 16: Related strains with flagellar locus mutation (M).
	*flaB*	C(48,49)	0		
	*fliD*	C(48,49)	0		
	*flgE*	C(48,49)	0		
	*flgK*	C(48,49)	0		
	*flgL*	C(48,49)	0		
17	*flaA*	ACD(50,51;52;57)	0		A(51) and ACD(50;52;57): Related strains with *flgL* loci mutations. Pt 17: Related strains with flagellar loci mutations (M).
	*flaB*	ACD(50,51;52;57)	0		
	*fliD*	ACD(50,51;52;57)	0		
	*flgE*	ACD(50,51;52;57)	0		
	*flgK*	ACD(50,51;52;57)	0		
	*flgL*	A(51) vs. ACD(50;52;57)=19	0.76%	M	
21	*flaA*	A(63) vs. AC(64;65,66)=25	1.63%	R	A(63) and AC(64;65,66): Related strains with *flaA* recombination. Pt 21: Related strains with *flaA* recombination (R), suggestive of superinfection by undetected, uncultured unrelated donor strain (s).
	*flaB*	AC(63,64;65,66)	0		
	*fliD*	AC(63,64;65,66)	0		
	*flgE*	AC(63,64;65,66)	0		
	*flgK*	AC(63,64;65,66)	0		
	*flgL*	AC(63,64;65,66)	0		
22	*flaA*	AC(70,71;72,96)	0		AC(70,71;72) and C(96) related strains showing a single mutation in each of the *fliD*, *flgE* and *flgL* gene loci. Pt 22: related strains exhibiting a single mutation in each of the three flagellin gene loci (M).
	*flaB*	AC(70,71;72,96)	0		
	*fliD*	AC(70,71;72) vs. C(96)=1	0.050%	M	
	*flgE*	AC(70,71;72) vs. C(96)=1	0.047%	M	
	*flgK*	AC(70,71;72,96)	0		
	*flgL*	AC(70,71;72) vs. C(96)=1	0.040%	M	
23	*flaA*	AC(73,74;75,76)	0		A(73,74) and C(75), A(73,74) and C(76): Related strains with *flgE* locus mutations. C(75) and C(76): Related strains with dual *flgE* locus mutations. Pt 23: Related strains with flagellar locus mutation profiles (M).
	*flaB*	AC(73,74;75,76)	0		
	*fliD*	AC(73,74;75,76)	0		
	*flgE*	A(73,74) vs. C(75)=1 C(75) vs. C(76)=2 A(73,74) vs. C(76)=1	0.047% 0.093% 0.047%	M M M	
	*flgK*	AC(73,74;75,76)	0		
	*flgL*	AC(73,74;75,76)	0		
25	*flaA*	CD(81,82;83,84)	0		CD(81,82;83) and D(84): Related strains with *flgK* locus mutation. Pt 25: Related strains with flagellar locus mutation (M).
	*flaB*	CD(81,82;83,84)	0		
	*fliD*	CD(81,82;83,84)	0		
	*flgE*	CD(81,82;83,84)	0		
	*flgK*	CD(81,82;83) vs D(84)=1	0.055%	M	
	*flgL*	CD(81,82;83,84)	0		
26	*flaA*	A(85) vs. A(86)=1	0.065%	M	A(85) and A(86): Related strains with *flaA* locus mutation. Pt 26: Related strains with flagellar locus mutation (M).
	*flaB*	A(85,86)	0		
	*fliD*	A(85,86)	0		
	*flgE*	A(85,86)	0		
	*flgK*	A(85,86)	0		
	*flgL*	A(85,86)	0		

Pt, patient; CDS, count of differential sites in aligned gene sequences from *H. pylori* mixed infections; CGEP, correlation of gene expression profiles among isolates from individual patients; EM, mechanistic explanation of *H. pylori* mixed infections in single-patient cases; U, unrelatedness; M, mutation; R, recombination; CM, convergent mutations; S, superinfection; SMCM, superinfection, mutation, and convergent mutations; SMR, superinfection, mutation, and recombination.

### 3.5 Housekeeping gene analysis

Analysis of 7 housekeeping genes in 27 patients identified mixed infections in 40.7% (11/27) patients. Gene-specific SDR reference ranges (5th–95th percentiles) were also established (Table 3). Cluster analysis distinguished low-proportion single-, triple-, and five-gene alterations (Cluster 1) from a highly frequent seven-gene alteration pattern (68.3%, 28/41) that constituted Cluster 2 (Figure 3 and Supplementary Table 5; Equation 2). SDR profiling attributed mixed infections to four mechanisms: mutation in 27.3% (3/11), recombination in 18.2% (2/11), combined mutation–recombination in 18.2% (2/11), and superinfection-associated mechanisms (S/SR/SCM) in 36.4% (4/11) of patients. Superinfection-associated mechanisms include convergent mutations in *mutY* (Table 6). Notably, Patient h25 represented a triple superinfection case with AD(H125–H138) and two additional unrelated corpus strains, C(H139) and C(H140) (Table 6).

**Figure 3:**
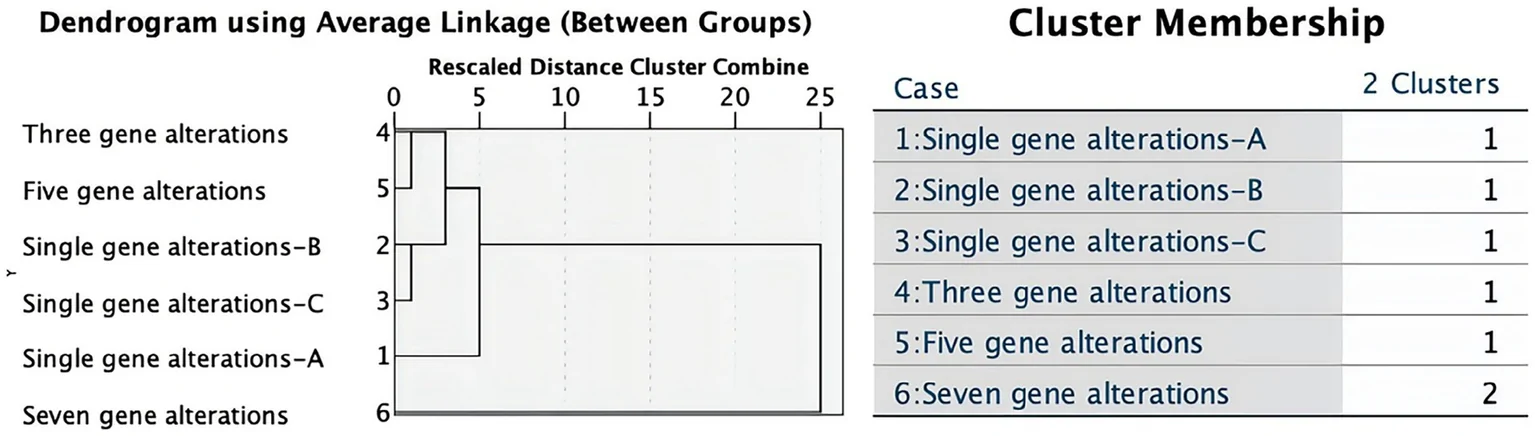
Clustering of housekeeping gene alterations in *H. pylori*.

**Table 6:** Mechanistic analysis of mixed *H. pylori* infections in the H group using the SDR method.

Pt	Gene	CDS	SDR	CGEP	EM
h1	*atpA*	A(H13) vs. CD(H21;H10)=6	0.96%	R	A(H13) and CD(H21;H10): Related strains demonstrating *atpA* recombination. AD(H13;H10) and C(H21): Related strains with *efp* locus mutations and *trpC* single-locus mutation. Pt h1: Related strains demonstrating *atpA* recombination coupled with *efp*/*trpC* locus mutations (MR), suggestive of superinfection by undetected, uncultured unrelated donor strain(s).
	*efp*	AD(H13;H10) vs. C(H21)=4	0.99%	M	
	*mutY*	ACD(H13;H21;H10)	0		
	*ppa*	ACD(H13;H21;H10)	0		
	*trpC*	AD(H13;H10) vs. C(H21)=1	0.22%	M	
	*ureI*	ACD(H13;H21;H10)	0		
	*yphC*	ACD(H13;H21;H10)	0		
h4	*atpA*	AD(H20;H19) vs. C(H18)=9	1.44%	R	AD(H20;H19) and C(H18): Related strains demonstrating *atpA* recombination. Pt h4: Related strains with *atpA* recombination (R), suggestive of superinfection by undetected, uncultured unrelated donor strain(s).
	*efp*	ACD(H20;H18;H19)	0		
	*mutY*	ACD(H20;H18;H19)	0		
	*ppa*	ACD(H20;H18;H19)	0		
	*trpC*	ACD(H20;H18;H19)	0		
	*ureI*	ACD(H20;H18;H19)	0		
	*yphC*	ACD(H20;H18;H19)	0		
h5	*atpA*	A(H23) vs. C(H42)=16	2.55%	U	A(H23) and C(H42): Unrelated strains. Pt h5: Superinfection involving unrelated strains (S).
	*efp*	A(H23) vs. C(H42)=11	2.71%	U	
	*mutY*	A(H23) vs. C(H42)=17	4.05%	U	
	*ppa*	A(H23) vs. C(H42)=6	1.55%	U	
	*trpC*	A(H23) vs. C(H42)=116	25.66%	U	
	*ureI*	A(H23) vs. C(H42)=6	1.04%	U	
	*yphC*	A(H23) vs. C(H42)=19	3.73%	U	
h11	*atpA*	A(H17) vs. C(H9)=3	0.48%	M	A(H17) and C(H9): Related strains with *atpA* locus mutations. Pt h11: Related strains with *atpA* locus mutation (M).
	*efp*	A(H17) vs. C(H9)=0	0		
	*mutY*	A(H17) vs. C(H9)=0	0		
	*ppa*	A(H17) vs. C(H9)=0	0		
	*trpC*	A(H17) vs. C(H9)=0	0		
	*ureI*	A(H17) vs. C(H9)=0	0		
	*yphC*	A(H17) vs. C(H9)=0	0		
h13	*atpA*	ACD(H86;H87;H52)	0		A(H86) and CD(H87;H52): Related strains with *yphC* locus mutations. Pt h13: Related strains with *yphC* recombination (R), suggestive of superinfection by undetected, uncultured unrelated donor strain(s).
	*efp*	ACD(H86;H87;H52)	0		
	*mutY*	ACD(H86;H87;H52)	0		
	*ppa*	ACD(H86;H87;H52)	0		
	*trpC*	ACD(H86;H87;H52)	0		
	*ureI*	ACD(H86;H87;H52)	0		
	*yphC*	A(H86) vs. CD(H87;H52)=15	2.95%	R	
h14	*atpA*	ACD(H92;H53;H93)	0		AC(H92;H53) and D(H93): Related strains with *mutY* single-locus mutation. Pt h14: Related strains with single-locus mutation (M).
	*efp*	ACD(H92;H53;H93)	0		
	*mutY*	AC(H92;H53) vs. D(H93)=1	0.24%	M	
	*ppa*	ACD(H92;H53;H93)	0		
	*trpC*	ACD(H92;H53;H93)	0		
	*ureI*	ACD(H92;H53;H93)	0		
	*yphC*	ACD(H92;H53;H93)	0		
h15	*atpA*	AD(H55,H56,H59,H60,H61,H62,H97;H63,H64) vs. AD(H57,H58;H65,H66,H68,H69,H70)=8	1.28%	R	AD(H55,H56,H59,H60,H61,H62,H97;H63,H64) and AD(H57,H58;H65,H66,H68,H69,H70): Related strains with *atpA* gene recombination (R). AD(H55,H56,H59,H60,H61,H62,H97;H63,H64) and D(H67): Related strains with *atpA* gene recombination (R). AD(H57,H58;H65,H66,H68,H69,H70) and D(H67): Related strains with *atpA* locus mutation (M). Pt h15: Related strains demonstrating both housekeeping gene recombination and single-locus mutation (MR), suggestive of superinfection by undetected, uncultured unrelated donor strain(s).
		AD(H57,H58;H65,H66,H68,H69,H70)vs. D(H67)=1	0.16%	M	
		AD(H55,H56,H59,H60,H61,H62,H97;H63,H64) vs. D(H67)=7	1.12%	R	
	*efp*	AD(H55,H56,H57,H58,H59,H60,H61,H62,H97;H63,H64,H65,H66,H67,H68,H69,H70)	0		
	*mutY*	AD(H55,H56,H57,H58,H59,H60,H61,H62,H97;H63,H64,H65,H66,H67,H68,H69,H70)	0		
	*ppa*	AD(H55,H56,H57,H58,H59,H60,H61,H62,H97;H63,H64,H65,H66,H67,H68,H69,H70)	0		
	*trpC*	AD(H55,H56,H57,H58,H59,H60,H61,H62,H97;H63,H64,H65,H66,H67,H68,H69,H70)	0		
	*ureI*	AD(H55,H56,H57,H58,H59,H60,H61,H62,H97;H63,H64,H65,H66,H67,H68,H69,H70)	0		
	*yphC*	AD(H55,H56,H57,H58,H59,H60,H61,H62,H97;H63,H64,H65,H66,H67,H68,H69,H70)	0		
h16	*atpA*	A(H75) vs. A(H71,H72,H73,H74,H76,H77,H78)=6	0.96%	R	A(H75) and A(H71,H72,H73,H74,H76,H77, H78): Related strains with *atpA* gene recombination (R). A(H75) and C(H79,H80,H81,H82,H83,H84, H85): unrelated strains. A(H71,H72,H73,H74,H76,H77, H78) and C(H79,H80,H81,H82,H83,H84, H85): unrelated strains. Pt h16: Superinfection involving both unrelated and related strains, manifesting SR patterns.
		A(H75)vs. C(H79,H80,H81,H82,H83,H84,H85)=12	1.91%	U	
		A(H71,H72,H73,H74,H76,H77,H78)vs. C(H79, H80,H81,H82,H83,H84,H85)=8	1.28%	U	
	*efp*	A(H71,H72,H73,H74,H75,H76,H77,H78)vs. C(H79,H80,H81,H82,H83,H84,H85)=14	3.46%	U	
	*mutY*	A(H71,H72,H73,H74,H75,H76,H77,H78)vs. C(H79,H80,H81,H82,H83,H84,H85)=12	2.86%	U	
	*ppa*	A(H71,H72,H73,H74,H75,H76,H77,H78)vs. C(H79,H80,H81,H82,H83,H84,H85)=7	1.81%	U	
	*trpC*	A(H71,H72,H73,H74,H75,H76,H77,H78)vs. C(H79,H80,H81,H82,H83,H84,H85)=14	3.10%	U	
	*ureI*	A(H71,H72,H73,H74,H76,H77,H78)vs. C(H79,H80,H81,H82,H83,H84,H85)=15	2.59%	U	
	*yphC*	A(H71,H72,H73,H74,H75,H76,H77,H78)vs. C(H79,H80,H81,H82,H83,H84,H85)=13	2.55%	U	
h24	*atpA*	A(H113,H114,H115,H116,H121,H122,H123,H124) vs. C(H117,H118,H119,H120)=12	1.91%	U	A(H113,H114,H115,H116,H121,H122,H123, H124) and C(H117,H118,H119, H120): Unrelated strains. Pt h24: Superinfection involving only unrelated strains (S).
	*efp*	A(H113,H114,H115,H116,H121,H122,H123,H124) vs. C(H117,H118,H119,H120)=14	3.5%	U	
	*mutY*	A(H113,H114,H115,H116,H121,H122,H123,H124) vs. C(H117,H118,H119,H120)=12	2.86%	U	
	*ppa*	A(H113,H114,H115,H116,H121,H122,H123,H124) vs. C(H117,H118,H119,H120)=7	1.81%	U	
	*trpC*	A(H113,H114,H115,H116,H121,H122,H123,H124) vs. C(H117,H118,H119,H120)=14	3.10%	U	
	*ureI*	A(H113,H114,H115,H116,H121,H122,H123,H124) vs. C(H117,H118,H119,H120)=15	2.59%	U	
	*yphC*	A(H113,H114,H115,H116,H121,H122,H123,H124) vs. C(H117,H118,H119,H120)=13	1.91%	U	
h25	*atpA*	AD(H125,H126,H127,H128,H129,H130,H131,H132;H133,H134,H135,H136,H137,H138) *vs.* C(H139)=11	1.75%	U	AD(H125,H126,H127,H128,H129, H130,H131,H132;H133,H134, H135,H136,H137,H138) and C(H139): Unrelated strains. AD(H125,H126,H127,H128,H129, H130,H131,H132;H133,H134, H135,H136,H137,H138) and C(H140): Unrelated strains. C(H139) and C(H140): Unrelated strains exhibiting convergent mutations in the *mutY* locus. Pt h25: Superinfection caused by unrelated strains with or without *mutY* mutations, resulting in SCM patterns.
		AD(H125,H126,H127,H128,H129,H130,H131,H132;H133,H134,H135,H136,H137,H138) *vs.* C(H140)=8	1.28%	U	
		C(H139) vs. C(H140)=11	1.75%	U	
	*efp*	AD(H125,H126,H127,H128,H129,H130,H131,H132;H133,H134,H135,H136,H137,H138) vs. C(H139)=7	1.73%	U	
		AD(H125,H126,H127,H128,H129,H130,H131,H132;H133,H134,H135,H136,H137,H138) vs. C(H140)=9	2.22%	U	
		C(H139) vs. C(H140)=6	1.48%	U	
	*mutY*	AD(H125,H126,H127,H128,H129,H130,H131,H132;H133,H134,H135,H136,H137,H138) vs. C(H139)=12	2.86%	U	
		AD(H125,H126,H127,H128,H129,H130,H131,H132;H133,H134,H135,H136,H137,H138) vs. C(H140)=11	2.62%	U	
		C(H139) vs. C(H140)=5	1.19%	CM	
	*ppa*	AD(H125,H126,H127,H128,H129,H130,H131,H132;H133,H134,H135,H136,H137,H138) vs. C(H139)=5	1.29%	U	
		AD(H125,H126,H127,H128,H129,H130,H131,H132;H133,H134,H135,H136,H137,H138) vs. C(H140)=3	0.78%	U	
		C(H139) vs. C(H140)=6	1.55%	U	
	*trpC*	AD(H125,H126,H127,H128,H129,H130,H131,H132;H133,H134,H135,H136,H137,H138) vs. C(H139)=13	2.88%	U	
		AD(H125,H126,H127,H128,H129,H130,H131,H132;H133,H134,H135,H136,H137,H138) vs. C(H140)=9	1.99%	U	
		C(H139) vs. C(H140)=16	3.54%	U	
	*ureI*	AD(H125,H126,H127,H128,H129,H130,H131,H132;H133,H134,H135,H136,H137,H138) vs. C(H139)=6	1.04%	U	
		AD(H125,H126,H127,H128,H129,H130,H131,H132;H133,H134,H135,H136,H137,H138) vs. C(H140)=4	0.69%	U	
		C(H139) vs. C(H140)=6	1.04%	U	
	*yphC*	AD(H125,H126,H127,H128,H129,H130,H131,H132;H133,H134,H135,H136,H137,H138) vs. C(H139)=12	2.36%	U	
		AD(H125,H126,H127,H128,H129,H130,H131,H132;H133,H134,H135,H136,H137,H138) vs. C(H140)=14	2.75%	U	
		C(H139) vs. C(H140)=7	1.38%	U	
h27	*atpA*	A(H156) vs. A(H160)=3	0.48%	M	Related strains, exhibiting the following mutant patterns: Single-locus mutations: AC(H146-H149,H155,H157-H160;H151,H162,H163) and C(H150,H152-H154,H161,H164): *efp*; A(H156) and AC(H146,H149,H155,H157-H160;H150-H154,H161-H164): *ppa*;A(H147,H156) and AC(H146,H148,H149,H155,H157-H160;H150-H154,H161- H164): *ureI*. Dual-locus mutations: A(H160) and AC(H146-H149,H155,H157-H159;H150-H154,H161-H164): *atpA*; A(H156) and AC(H146-H149,H155,H157- H159-H163): *efp*. Multilocus mutations: A(H156) and A(H160): *atpA*; A(H156) and AC(H146-H159;H150-H154, H161-H164): *atpA*; A(H156) and C(H150-H154,H161,H164): *efp*; A(H156) and AC(H146-H149,H155,H157-H160;H150-H154,H161-H164): *trpC*. Pt h27: Related strains with nine locus-specific mutation patterns (M).
		A(H156) vs AC(H146,H147,H148,H149,H155,H157,H158,H159;H150,H151,H152,H153,H154,H161,H162,H163,H164)=3	0.48%	M	
		A(H160) vs. AC(H146,H147,H148,H149,H155,H157,H158,H159;H150,H151,H152,H153,H154,H161,H162,H163,H164)=2	0.32%	M	
	*efp*	A(H156) vs. AC(H146,H147,H148,H149,H155,H157,H158,H159,H160;H151,H162,H163)=2	0.49%	M	
		A(H156) vs. C(H150,H152,H153,H154,H161,H164)=3	0.74%	M	
		AC(H146,H147,H148,H149,H155,H157,H158,H159,H160;H151,H162,H163) vs. C(H150,H152,H153,H154,H161,H164)=1	0.25%	M	
	*mutY*	AC(H146,H147,H148,H149,H155,H156,H157,H158,H159,H160;H150,H151,H152,H153,H154,H161,H162,H163,H164)	0		
	*ppa*	A(H156) vs. AC(H146,H147,H148,H149,H155,H157,H158,H159,H160;H150,H151,H152,H153,H154,H161,H162,H163,H164) =1	0.26%	M	
	*trpC*	A(H156) vs. AC(H146,H147,H148,H149,H155,H157,H158,H159,H160;H150,H151,H152,H153,H154,H161,H162,H163,H164)=5	1.11%	M	
	*ureI*	A(H147,H156) vs. AC(H146,H148,H149,H155,H157,H158,H159,H160;H150,H151,H152,H153,H154,H161,H162,H163,H164) =1	0.17%	M	
	*yphC*	AC(H146,H147,H148,H149,H155,H156,H157,H158,H159,H160;H150,H151,H152,H153,H154,H161,H162,H163,H164)	0		

Pt, patient; CDS, count of differential sites in aligned gene sequences from *H. pylori* mixed infections; CGEP, correlation of gene expression profiles among isolates from individual patients; EM, mechanistic explanation of *H. pylori* mixed infections in single-patient cases; U, unrelatedness; M, mutation; R, recombination; CM, convergent mutations; S, superinfection; SR, superinfection and recombination; SCM, superinfection and convergent mutations.

### 3.6 Phylogenetic validation and geographical divergence assessment

Phylogenetic trees (Figures 4A–F, 5A–G) validated the SDR framework; shared nodes corresponded to sequence identity (SDR = 0; Table 3 and Supplementary Table 6), short branches reflected mutational changes, and long branches denoted genetic unrelatedness. However, a single anomaly illustrated the limitations of phylogenetic analysis alone; strain H156 (*ppa*) displayed a long branch despite minimal genetic divergence (0.26% SDR; Figure 5D), underscoring the need for quantitative SDR-based validation to avoid misclassification.

**Figure 4:**
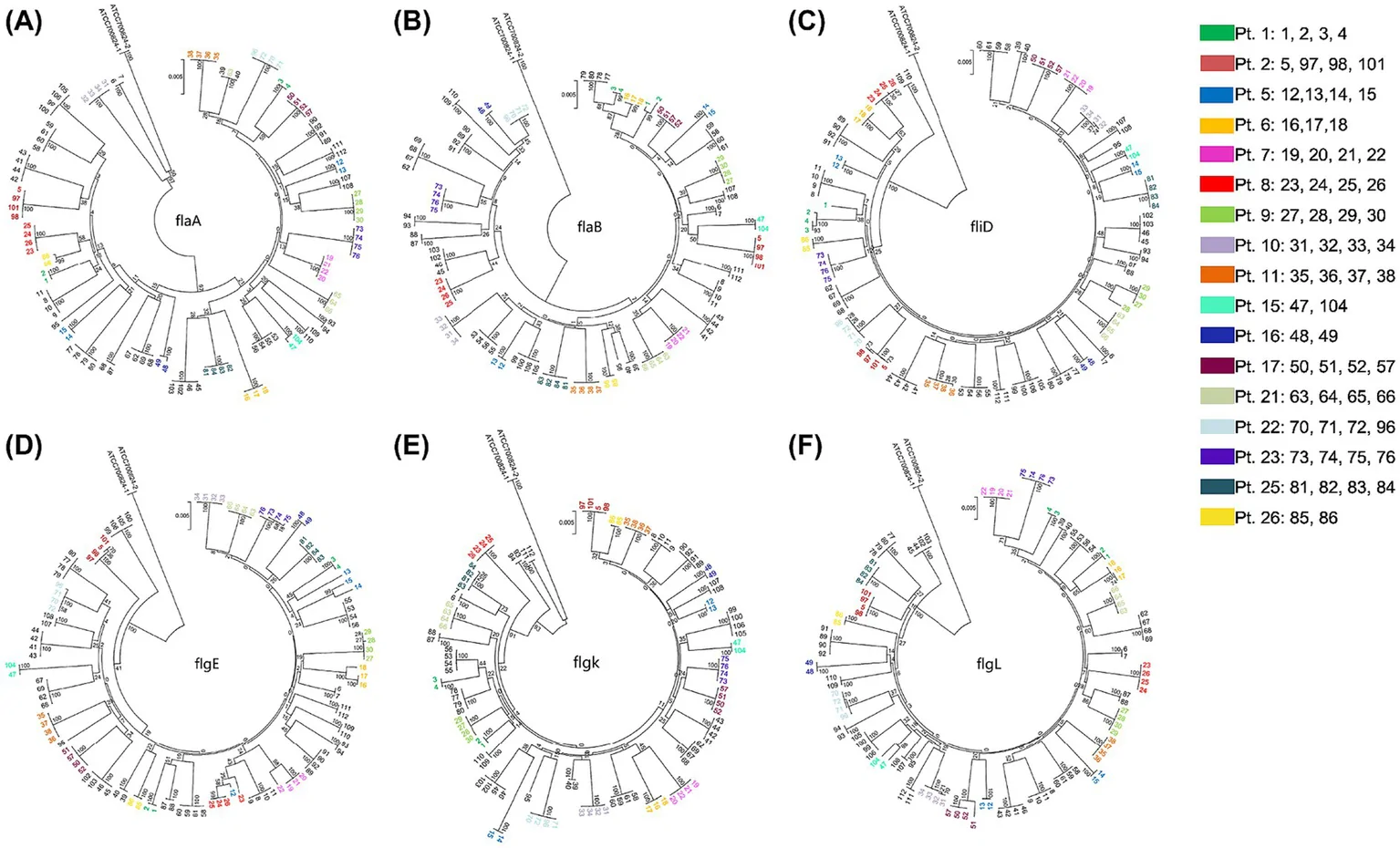
Phylogeny of *H. pylori* flagellar genes. **(A–F)** Strain numbers are color-coded by patient, with 17 distinct colors used to differentiate between mixed infection cases. Genetic relationships between strains from the same patient are illustrated; the relationship between two representative strains **(a,b)** is shown as an example (Supplementary material, pp. 8–10).

**Figure 5:**
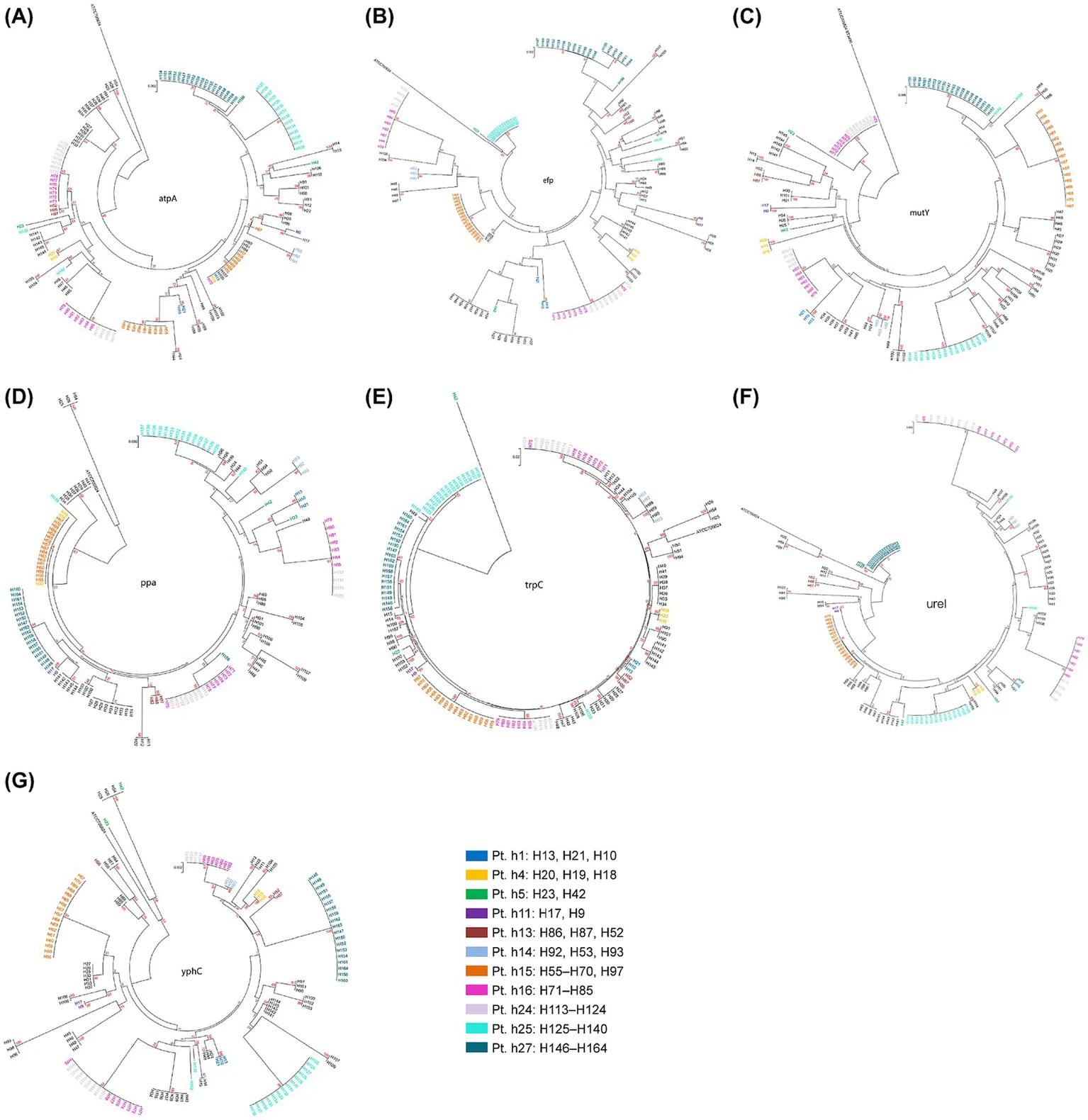
Phylogeny of *H. pylori* housekeeping genes. **(A–G)** Strain numbers are color-coded using 11 distinct colors, with identical colors indicating strains from the same patient and different colors indicating strains from different patients. The genetic relationship between two representative strains **(a,b)** from a single patient is shown (Supplementary material, pp. 11–13).

Geographical divergence was determined using gene-specific regional SDR thresholds, which were defined as SDR values exceeding the established reference range for at least three genes. Reference strains ATCC 700824 (Supplementary Tables 7, 8) and H54 (exogenous ST3699; Supplementary Table 9) met this criterion, confirming their non-local origins. Strain H42 exhibited markedly elevated *trpC* SDR values (23.89–25.66%; Figure 5E and Supplementary Table 10) exceeding the regional reference range (1.77–24.30%), indicating heterologous recombination (interspecies horizontal gene transfer).

In parallel, the phylogenetic patterns independently confirmed infection mechanisms. Superinfection-associated strains were separated by long branches across all phylogenetic trees, visually corroborating superinfection subtypes, such as SMR (Patient 5) and SMCM/SCM, along with their respective convergent mutations.

### 3.7 Statistical power and precision of prevalence estimates

The primary analyses had adequate statistical power. The key comparison, superinfection prevalence in flagellar (6.1%) versus housekeeping (14.8%) genes, achieved 0.82 power, above the usual 0.80 cutoff. Detecting the dominant mutation pattern in flagellar genes (82.4% vs. 50% null) had power well above 0.99. The 95% confidence intervals were 34.2–68.8% for mixed infection via flagellar genes and 4.2–34.9% for superinfection via housekeeping genes. Full parameters are in Supplementary Table 3.

### 3.8 Discovery of *trpC* heterologous recombination in strain H42

SDR analysis identified the *trpC* locus in strain H42 as a candidate for heterologous recombination. The ULR was 13.73 (24.30/1.77), exceeding the threshold for the initial screen. Confirmatory assessment showed that the focal SDR range (23.89–25.66%) exceeded both the local reference upper bound (24.30%) and the external reference range for ATCC 700824 (6.64–8.50%; Table S8), thereby satisfying the criteria in Section 2.7.1.

BLASTn analysis of the H42 *trpC* sequence against the NCBI nucleotide database identified *Maribacter* sp. as the top hit (98% identity). Among non-*H. pylori* species, *H. cetorum* (99% identity to partial *trpC*) and *H. acinonychis* (99% identity) showed the highest sequence identities, with no *H. pylori* match exceeding these values.

For phylogenetic validation, a dataset of 241 sequences was assembled from sequences available in the NCBI nucleotide database: 151 sequences from this study, the reference strain ATCC 700824, and 89 sequences retrieved by BLASTn (81 *H. pylori*, 5 other *Helicobacter* species, 2 predicted *Temnothorax* spp., and 1 *Maribacter* sequence). Maximum-likelihood reconstruction placed the H42 *trpC* sequence outside the *H. pylori* clade, where it instead clustered with other *Helicobacter* species (Figure 6). The concordance among SDR metrics, BLASTn sequence top-hit identities, and phylogenetic placement supports the hypothesis that this *trpC* allele was acquired through interspecies horizontal gene transfer and validates the capacity of the framework to detect heterologous recombination events.

**Figure 6:**
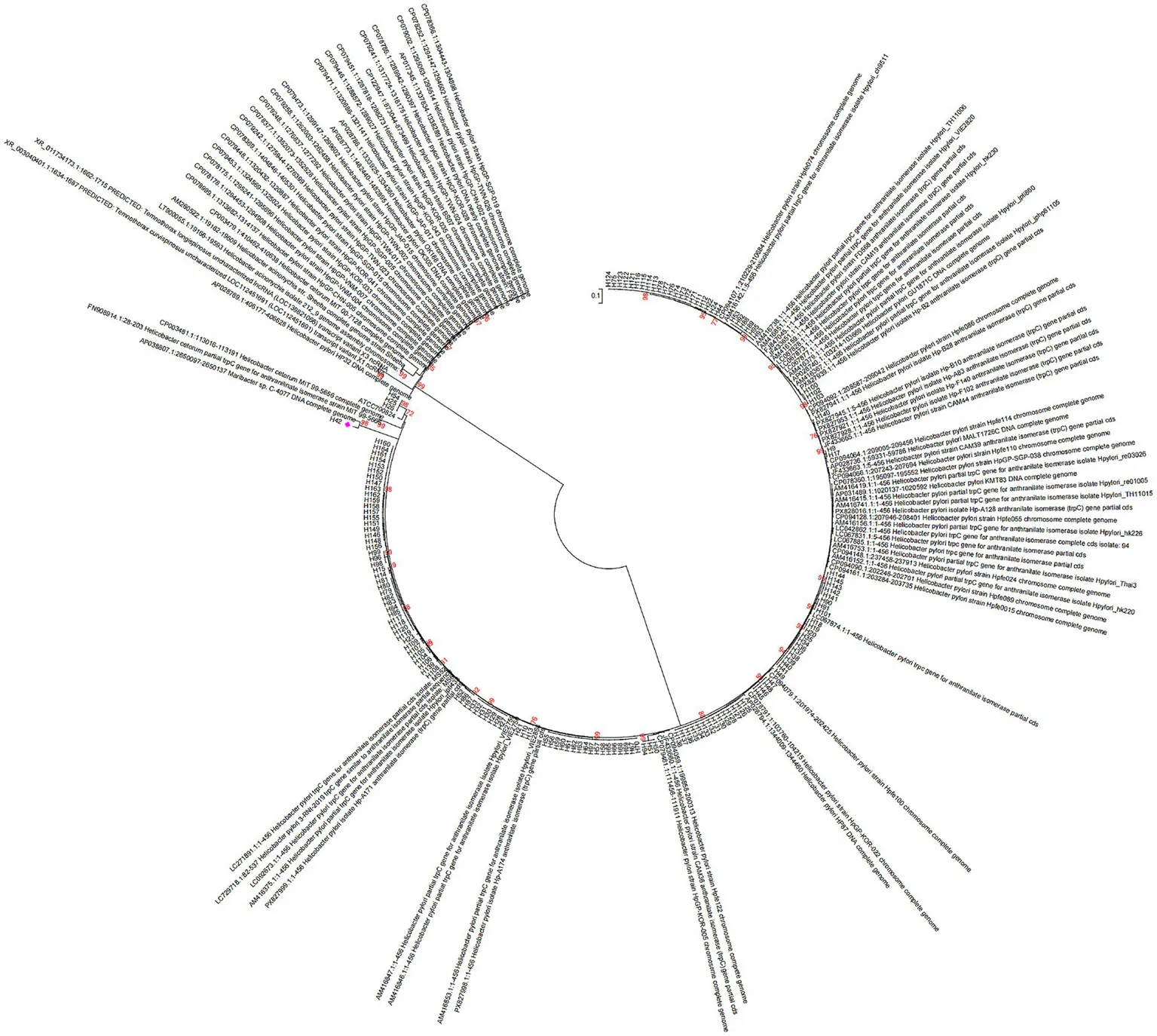
Maximum likelihood phylogeny of *trpC* sequences including study isolates, reference strain ATCC 700824, and non-*H. pylori* species identified by BLASTn analysis. The H42 sequence (purple diamond) occupies a basal position relative to the *H. pylori* clade, branching with *H. cetorum* and *H. acinonychis*. This topology supports heterologous recombination. Bootstrap values >50% are shown at nodes. Scale bar: substitutions per site.

## 4 Discussion

The culture system developed in this study recovers *H. pylori* from gastroduodenal biopsies at 94.1–100% even when the specimens were processed with delay (Zhang et al., 2023). All isolates were obtained using our patented dilution-based single-colony technique, which avoids the problem of conventional streaking. Standard streaking results in poor single-colony isolation and a progressive loss of viability in serial subcultures. In contrast, our method routinely yielded a sufficient number of single colonies for downstream studies.

Serial subcultures of *H. pylori* cause genotypic loss (Ren et al., 2012) and subculture failure (Arévalo-Jaimes et al., 2019), compromising mixed infection studies. Our method shortens the culture period and reduces genetic drift during successive subcultures, thereby preserving the mixed strain population structure in individual hosts (Zhang et al., 2023). It minimizes detection bias and strain misclassification from poor recovery or subculture-associated genetic changes and supports quantitative SDR profiling. Building on this reliable culture platform, we developed an SDR framework, a dedicated quantitative analytical tool that categorizes the four core mechanisms underlying *H. pylori* mixed infections: mutation (M), recombination (R), combined mutation–recombination (MR), and superinfection (S). By integrating epidemiological criteria for defining genetically unrelated strains with mathematical modeling and multigene comparative profiling, this framework establishes gene-specific SDR threshold cutoffs to accurately distinguish mutational variation, homologous recombination, and exogenous genetic unrelatedness. Notably, the R and MR patterns identified via SDR analysis may indicate occult superinfection events, wherein unrelated donor strains escape detection due to the inherent limitations of isolation schemes or insufficient colony sampling depth.

SDR-detected recombination involved conserved flagellar genes *flgE* and *flaA* and housekeeping genes *atpA* and *yphC*. These loci differ from recombination-prone loci reported in early *H. pylori* studies, notably *cag* and *ppsA* (HP0121) (Kersulyte et al., 1999), which are linked to strain-specific virulence and metabolic functions. Thus, the SDR framework broadens the known spectrum of recombination targets in this pathogen, implicating conserved structural and metabolic genes in the within-host evolutionary dynamics.

As an illustrative example, Patient h25 was processed using Isolation Scheme c, with eight single colonies randomly selected from both the gastric antrum and corpus, and two genetically unrelated strains were successfully identified from corpus specimens. In comparison, Patient 21 (R) had only two randomly selected single colonies sampled from the antrum and corpus, whereas patients h1 (MR), h4 (R), and h13 (R) were processed using Isolation Scheme a, with only one random colony isolated from the antrum, corpus, and duodenal bulb, respectively. The absence of detectable unrelated strains in these latter patients may be partially attributed to insufficient sampling intensity, although such limitations cannot fully explain the observed evolutionary patterns. Consistent with this interpretation, Mendoza-Elizalde et al. (2019) reported two enrolled patients from whom 20 antral colonies were isolated before treatment, with 2 distinct unrelated strains identified in each patient. Collectively, these findings confirm that the recovery efficiency of unrelated *H. pylori* strains is positively correlated with the number of colonies analyzed. This SDR-based analytical strategy resolves the long-standing ambiguity and subjective judgment that have hampered conventional genotyping accuracy in differentiating intrahost bacterial evolution from superinfection (Carroll et al., 2004).

A key finding of this study is the empirical evidence of convergent mutations in *fliD* (flagellar) and *mutY* (housekeeping) genes within superinfection–mutation (SM) mechanisms, indicating adaptive evolution within the gastric niche. Although *H. pylori* convergent mutations have been predicted using bioinformatics simulations (Chattopadhyay et al., 2018), specific case characterization is lacking. We report the first clinical isolates with convergent mutations in *fliD* and *mutY*, providing direct evidence of the niche-specific adaptive evolution of this pathogen.

We further uncovered fundamental divergence in the evolutionary dynamics of flagellar and housekeeping genes; mutations dominated flagellar genes (82.3% of cases), whereas housekeeping genes showed a balanced distribution of M, R, and S mechanisms. Cluster analysis corroborated this dichotomy by linking multigene alteration profiles (Cluster 2) to superinfection, in contrast to the limited changes (Cluster 1) arising from founding strain diversification (Mendoza-Elizalde et al., 2019; Carroll et al., 2004; Wilkinson et al., 2022). Notably, we identified a case of triple-strain superinfection (Patient h25), which represents the first documented evidence of three genetically distinct, unrelated strains concurrently colonizing a single host, further illustrating the complexity of within-host strain dynamics.

The SDR framework overcomes the critical limitations of earlier methods (e.g., DNA fingerprinting (Raaf et al., 2017) and MLST-based phylogenies (Zhang et al., 2023)), which cannot reliably separate mutations from recombination or link genetic data to the epidemiological context. For instance, strains from Patient h27 (ST3720, ST3724, ST3742, ST3743, and ST3749) previously classified as unrelated (Zhang et al., 2023) were re-classified as genetically related using SDR analysis. This conclusion was supported by convergent evidence: SDR profiles indicated a mutation-driven mechanism, and phylogenetic trees confirmed close relatedness through short branch lengths. Such concordance validates the SDR approach while highlighting that phylogenies alone lack the quantitative resolution to mechanistically distinguish mutations from strain-unrelatedness. The single discrepancy involving the *ppa* gene (SDR = 0.26%) underscores the need for multigene validation to avoid misclassification. Furthermore, while a prior study used neighbor-net graphs to infer recombination (Zhang et al., 2023), it did not quantify its prevalence or delineate specific genetic changes, which are limitations directly addressed by the quantitative thresholds of the SDR framework.

Our findings have several important clinical implications. Unrelated strains exhibited a consistent corpus-restricted colonization pattern across all superinfection cases, absent from the antrum and duodenal bulb, likely reflecting the antral niche occupation by the founding strain (Jackson et al., 2020). This spatial segregation of genetically distinct strains substantially challenges current treatment paradigms (Malfertheiner et al., 2022), which are based on the assumption of single-strain infection. Superinfecting strains may harbor unique antimicrobial resistance profiles, a well-documented contributor to heteroresistance (Rizvanov et al., 2019), suggesting that standard empirical therapeutic regimens risk failure owing to inadequate coverage of heterogeneous bacterial populations. Therefore, our findings challenge the long-standing practice of uniform treatment and emphasize the necessity for personalized eradication regimens. The SDR framework provides a reliable and practical criterion for superinfection screening, and profiling of at least three genes is required to eliminate the confounding effects of convergent mutations, with *flaB*, *flgK*, and *flaA* representing the optimal candidates because of their low CO values.

Incomplete anatomical sampling may also obscure superinfection. Patient h15, with an MR pattern, yielded nine antral and eight duodenal bulb colonies but no corpus isolates. The absence of corpus isolates likely led to missed superinfections because the unrelated strains were corpus-restricted. This highlights the need for combined antral and corpus sampling to identify superinfections accurately.

Unrelated strains exhibited two distinct corpus–antral distribution patterns in mixed infections. In the first pattern, observed in patients 1, 5, h5, h16, and h24, the antrum carried the founding strain and the corpus carried only unrelated strains. This pattern was previously documented by RAPD-PCR in 1 of 41 patients using Isolation Scheme a (Kim et al., 2003) and was detectable with isolation schemes a or c. In the second pattern, observed only in Patient h25, the antrum harbored the founding strain alone or together with its mutants, whereas unrelated strains were a minority in the corpus. This pattern requires Isolation Scheme c, as a sufficient number of single colonies must be analyzed to recover a minority of unrelated strains. Findings from Patient h25 suggest that at least eight single-colony isolates per site are required for both the antrum and corpus to detect this pattern. However, the predominant epidemiological pattern remains unknown.

A comparable pattern was reported by Salama et al. (2007), who applied RAPD and AFLP to analyze 16 isolates from 4 gastric sites (including the antrum and corpus) in 1 of 8 patients using Isolation Scheme c. Five antral isolates were uniformly Pattern A, while the remaining 11 isolates from other sites comprised four of Pattern A and seven of Pattern B. Patterns A and B were completely distinct, confirming that the seven isolates represented unrelated strains. This resembled our second pattern, although RAPD and AFLP differ fundamentally from sequence-based methods and lack epidemiological grounding for defining unrelated strains.

The utility of SDR extends beyond the pathogenicity of *H. pylori*. It enables precise characterization of probiotic engraftment, post-therapeutic recurrence, and mixed colonization in diverse microbial ecosystems (e.g., *M. tuberculosis* and gut commensals), addressing a central gap in microbiome research that low-resolution methods such as 16S rRNA sequencing cannot resolve. Additionally, the ability of SDR to determine geographic clustering (e.g., distinguishing hspEAsia from hspWAfrica strains) (Zhang et al., 2023) supports the epidemiological tracing of transmission routes, which is critical for designing targeted eradication programs. Our prevalence estimates align with those from East Asian cohorts, where multi-strain colonization is common.

Our study has several limitations. First, the SDR thresholds were derived from a regional cohort. Accurate assessment of gene evolution depends on reference ranges that capture local strain diversity; therefore, these thresholds are specific to our study region and cannot be applied to other populations. Although the framework can be recalibrated for different geographic settings, each target population requires independent threshold establishment and validation. Second, the reliance of this method on high base-call accuracy currently limits its direct application to whole-genome sequencing (Wilkinson et al., 2022; Cao et al., 2015), constraining its use in first-generation sequencing technologies (Zhang et al., 2023). Third, the F and H groups differed in disease severity and isolation scheme use. The F group, recruited earlier with more severe cases, mostly used scheme c (fixed 4 colonies), while the H group, recruited later with mostly gastritis, used scheme a (1–3 colonies) with selective high-intensity sampling. These differences make direct prevalence comparison difficult, though they do not affect the SDR framework itself, as thresholds were set independently within each group. Finally, the single-region design and lack of long-term follow-up limit how far we can generalize the findings and track the clinical course of superinfection.

Our findings resolve a key uncertainty in the field. Whereas Salama et al. (2007) speculated about a potential superinfection case and Mendoza-Elizalde et al. (2019) described genotypic patterns as “re-colonization,” our SDR framework now provides conclusive evidence that such scenarios represent definitive superinfections. Furthermore, the utility of the SDR framework extends beyond resolving within-host evolutionary mechanisms to enable the precise geographic clustering of *H. pylori* lineages. Although MLST-based phylogeography has been conventionally used for this purpose, our analysis provides a higher-resolution quantitative alternative (Vazirzadeh et al., 2022; Zhang et al., 2023). We identified a phylogenetically discordant ST3699 strain from a patient native to Ningbo, which exhibited SDR reference ranges for five housekeeping genes (*atpA, efp, mutY, ureI,* and *yphC*) that fell outside the spectrum characteristics of the local hspEAsia population (Zhang et al., 2023), thereby confirming its exogenous origin. This finding was corroborated by analysis of the reference *H. pylori* strain ATCC 700824 (Zhang et al., 2023), whose SDR ranges for both flagellar and housekeeping genes distinctly differed from those of the Ningbo strains.

Most notably, ULR-based screening identified *trpC* as a candidate for heterologous recombination with a ULR of 13.73, substantially exceeding the threshold of 10. Follow-up analysis revealed that *H. pylori* strain H42 exhibited an exceptionally high *trpC* SDR range (23.89–25.66%), extending beyond the regional reference bounds (1.77–24.30%). BLASTn and phylogenetic analysis bore out the SDR prediction. *Maribacter* sp. was the top hit (98% identity), and *Helicobacter* species other than *H. pylori* (*H. cetorum*, 99%; *H. acinonychis*, 99%) outscored every *H. pylori* match. The H42 *trpC* sequence sat outside the *H. pylori* clade. SDR flagged an outlier, BLAST found a closer match in another genus, and the tree placed H42 between *H. pylori* and other *Helicobacter* species. These three independent lines of evidence show that the framework can pick up inter-species horizontal gene transfer at nucleotide resolution, a step beyond what current genotyping methods offer. This is the first direct evidence of exogenous genetic material acquisition via heterologous recombination in this pathogen. Phylogenetic reconstruction and geographical divergence analyses independently supported this recombination event at the *trpC l*ocus in strain H42.

Previous studies on *H. pylori* recombination (Raymond et al., 2004; Falush et al., 2001; Chattopadhyay et al., 2018) have uniformly addressed homologous exchange between conspecific strains, with the rarity of heterologous recombination attributed to a relatively isolated gastric niche (Falush et al., 2001). However, *H. pylori*–colonized mucosa harbors diverse microbiota, including Enterobacteriaceae and other taxa, creating conditions that facilitate cross-species genetic transfer. The SDR framework moves beyond simply describing this phenomenon: it provides a quantitative, sequence-based criterion for identifying exogenous genetic material in clinical isolates. This discovery establishes the SDR framework as the first tool capable of detecting interspecies horizontal gene transfer in *H. pylori* at nucleotide resolution, a capability lacking in existing genotyping methods. By extending beyond intra-specific strain discrimination to the identification of exogenous genetic material from other bacterial species, the SDR framework uncovered an evolutionary process that was previously inaccessible using conventional approaches. Collectively, these findings demonstrate that SDR reference ranges provide a robust sequence-based tool that adds a critical population genetics and evolutionary dimension to *H. pylori* molecular epidemiology.

The SDR framework opens several avenues for future research. Priority should be given to: validating SDR thresholds across diverse global populations to establish universal baselines (Zhang et al., 2023); conducting clinical trials to evaluate whether SDR-guided antimicrobial susceptibility testing improves eradication rates in patients with mixed or superinfected strains (Hamze et al., 2025); functionally characterizing convergent mutations in *fliD* and *mutY* to elucidate their roles in niche adaptation; optimizing SDR for compatibility with whole-genome sequencing data to enhance scalability and resolution; systematically evaluating how isolation scheme and sampling intensity affect the detection of mixed infections and superinfection, with standardized protocols for future comparative studies; and assessing the long-term clinical consequences of superinfection, including its impact on treatment failure and disease progression. Beyond *H. pylori*, the establishment of universal SDR baselines will enable the application of this framework to other pathogens, such as *M. tuberculosis* (Sobkowiak et al., 2018; Chengalroyen et al., 2022; Liu et al., 2025), as well as to microbiome engineering efforts, where it may help elucidate strain-mixing dynamics in probiotic and commensal microbial communities.

Our primary findings are well supported by the statistical analysis. Post-hoc power was 0.82 for comparing superinfection rates between gene categories, and above 0.99 for detecting the dominant mutation pattern in flagellar genes. The 95% confidence intervals (34.2–68.8% for mixed infection; 4.2–34.9% for superinfection) are wide, as expected for a pilot study. These results support the mechanistic classifications reported here, but larger cohorts will be needed to refine estimates for rare events and establish reference ranges across different regions. Future multi-center studies should develop region-specific SDR reference ranges from locally isolated strains before applying the method in new geographic settings. Although a universal range would facilitate standardization, calibration against local strain collections may improve the accuracy of the method. We list multi-center validation as a priority.

## 5 Conclusion

In summary, we developed and validated a robust SDR framework that addresses a long-standing methodological bottleneck in *H. pylori* evolutionary research. For the first time, this quantitative tool enables systematic discrimination of mutations, homologous recombination, and superinfection during intrahost mixed infections, thereby overcoming the ambiguity and limited resolution inherent to conventional genotyping methods. Our findings document complex within-host infection dynamics, including the first reported clinical case of triple-strain concurrent superinfection, and reveal distinct evolutionary patterns between flagellar and housekeeping genes during chronic gastric colonization.

The SDR framework revealed two previously unrecognized evolutionary characteristics of *H. pylori*. First, nucleotide-level evidence of interspecies heterologous recombination at the *trpC* locus confirmed ongoing cross-species genetic exchange within the gastric niche. Second, niche-specific convergent mutations in *fliD* and *mutY* demonstrated adaptive genomic evolution driven by gastric environmental selection. These findings expand our current understanding of the *H. pylori* recombination spectrum and its adaptive evolutionary mechanism.

This study validates the use of the SDR framework for classifying mechanisms of *H. pylori* mixed infection. We identified superinfecting strains in select cases, including one triple-strain infection; however, the relationship between SDR-detected superinfection and resistance heterogeneity remains unclear. Whether genetically distinct co-colonizing strains exhibit different drug susceptibilities, and whether these differences are associated with their distribution within the stomach, can only be determined through prospective multi-colony testing. Until such data are available, SDR is best used as a research tool to identify complex infections for further investigation.

Clinically, our results pose a fundamental challenge to the conventional one-size-fits-all empirical treatment paradigm for *H. pylori* infections. The spatial segregation and genetic heterogeneity of the superinfecting strains provide a clear mechanistic basis for clinical heteroresistance and treatment failure, underscoring the urgent need for strain-specific personalized eradication strategies guided by precise strain identification and antimicrobial susceptibility testing. As a sequence-based quantitative tool, the SDR framework enriches *H. pylori* molecular epidemiology by providing valuable population-genetic and evolutionary insights.

SDR profiling further confirmed that samples displaying only R or MR patterns inherently harbored superinfecting strains, which are often missed because of inadequate single-colony recovery at each anatomical site or a lack of isolates from the gastric corpus. Therefore, combined biopsy sampling of the antrum and corpus with sufficient single-colony isolates is indispensable for the robust detection of superinfection events.

Because of its high resolution, excellent reproducibility, and broad expandability, the SDR method offers a reliable solution for distinguishing intrahost evolution from superinfection, tracing bacterial phylogeography, and interpreting complex microbial strain dynamics. This framework holds substantial promise for future applications in precision infectious disease surveillance, clinical therapeutic optimization, and broader microbiome and pathogen evolutionary research.

## Data Availability

The datasets presented in this study can be found in online repositories. The names of the repository/repositories and accession number(s) can be found at: The data (nucleotide sequences) generated in this study have been deposited in the NCBI GenBank database under accession numbers PQ035224–PQ035907 (flagellar genes) and PQ606662–PQ607725 (housekeeping genes).
