# Supplementary material for: A validated sequence difference ratio framework distinguishes intrahost evolution from superinfection in Helicobacter pylori mixed infections
Source: Front Microbiol. 2026 Oct 6;17:1948969. doi: 10.3389/fmicb.2026.1948969 (PMC13718763; doi:10.3389/fmicb.2026.1948969)
Supplement: Supplementary file 1 [file Supplementary_file_2.doc]

Supplementary Material

# 1 Supplementary Tables

Table S2. Primers used for the six flagellar genes.

| **Gene** | **Primer sequence (5′→3′)** | **AFL (bp)** | **AT (℃)** |
| --- | --- | --- | --- |
| *flaA* | *flaA*-1F-AATTTCAAACTTTAACCG | 1935 | 46.5 |
|  | *flaA*-1R-CAAAACTATCCAACACGA |  |  |
| *flaA* | *flaA*-2F-CCTTAGAGGAATGTTTTTTATGG | 1938 | 55 |
|  | *flaA*-2R-AGCTTCAAATTTCAGAGCGTTAG |  |  |
| *flaB* | *flaB*-F-GATTCTACGATAATAAGGTGCTTC | 1958 | 55 |
|  | *flaB*-R-ATAAGGGCGTGTTTGTTTCT |  |  |
| *fliD* | *fliD*-F-GAGCGTCTGAATGAAGAAATG | 2358 | 55 |
|  | *fliD*-R-ACTCACTCGGTTATGCTGGTA |  |  |
| *flgE* | *flgE*-1F-AAATCCGCAACCAATCAT | 2409 | 55 |
|  | *flgE*-1R-CACCAATACCGACCACGA |  |  |
| *flgE* | *flgE*-2F-CTTTAAAATCCGCAACCAAT | 2412 | 55 |
|  | *flgE*-2R-TCTTTCACCAATACCGACCA |  |  |
| *flgK* | *flgK*-F-CGTTTGCGGTTTCTGAAT | 2051 | 55 |
|  | *flgK*-R-ATAACACAAGCTTTACAAAACACTC |  |  |
| *flgL* | *flagL*-F-TTGCGTATGACATTTCTATTCC | 2858 | 55 |
|  | *flagL*-R-TTGCCCACGAAATAAAGG |  |  |

**Note:** AFL, amplified fragment length; AT, annealing temperature; ET, extension time. Owing to the high variability of *flaA* and *flgE*, frequent insertion–deletions and single-nucleotide polymorphisms are present, requiring two primer pairs for reliable amplification.

Table S3. Post-hoc power analysis parameters and results

| **Cohort** | **Parameter** | **Observed Value** | **95% CI** | **Test** | **Effect Size** | **Achieved Power** | **Note** |
| --- | --- | --- | --- | --- | --- | --- | --- |
| Flagellar (n=33) | Mixed infection prevalence | 51.5% (17/33) | 34.2–68.8% | Exact binomial | — | — | Clopper-Pearson |
| Housekeeping (n=27) | Mixed infection  prevalence | 40.7% (11/27) | 23.2–59.8% | Exact binomial | — | — | Clopper-Pearson |
| Flagellar (n=33) | Superinfection  prevalence | 6.1%  (2/33) | 0.7–20.2% | Exact binomial | — | — | Clopper-Pearson |
| Housekeeping (n=27) | Superinfection  prevalence | 14.8% (4/27) | 4.2–34.9% | Exact binomial | — | — | Clopper-Pearson |
| Between groups | Difference in superinfection prevalence | 8.70% | — | Two-proportion z-test | h=0.45 | 0.82 | G*Power 3.1.9.7; α = 0.05, two-tailed |
| Flagellar | Mutation-dominant pattern | 82.4% (14/17) | — | One-proportion z-test | — | >0.99 | vs. 50% null;  α = 0.05 |

Note: CI, confidence interval; —, not applicable. Clopper-Pearson exact method used for binomial confidence intervals; G*Power version 3.1.9.7 used for power calculations.

Table S4. Flagellar gene alteration patterns and proportions in *H. pylori* mixed infections.

| **PNGAE** | **Pt No.** | ***flaA*** | ***flaB*** | ***fliD*** | ***flgE*** | ***flgK*** | ***flgL*** |
| --- | --- | --- | --- | --- | --- | --- | --- |
| Single-gene alterations A | 11 | 0% | 0% | 2.9% (1/34) | 0% | 0% | 0% |
| Single-gene alterations B | 16,21,26 | 8.8% (3/34) | 0% | 0% | 0% | 0% | 0% |
| Single-gene alterations C | 7,8,23 | 0% | 0% | 0% | 8.8% (3/34) | 0% | 0% |
| Single-gene alterations D | 17, | 0% | 0% | 0% | 0% | 0% | 2.9% (1/34) |
| Single-gene alterations E | 25 | 0% | 0% | 0% | 0% | 2.9% (1/34) | 0% |
| Two-gene alterations A | 2,9 | 0% | 0% | 5.9% (2/34) | 5.9% (2/34) | 0% | 0% |
| Two-gene alterations B | 6 | 0% | 0% | 0% | 2.9% (1/34) | 0% | 2.9% (1/34) |
| Two-gene alterations C | 10 | 0% | 0% | 2.9% (1/34) | 0% | 0% | 2.9% (1/34) |
| Two-gene alterations D | 15 | 2.9% (1/34) | 0% | 0% | 0% | 0% | 2.9% (1/34) |
| Three-gene alterations | 22 | 0% | 0% | 2.9% (1/34) | 2.9% (1/34) | 0% | 2.9% (1/34) |
| Six-gene alterations | 1,5 | 5.9% (2/34) | 5.9% (2/34) | 5.9% (2/34) | 5.9% (2/34) | 5.9% (2/34) | 5.9% (2/34) |

**Note:** PNGAE patterns based on the number of gene alteration events. Proportion = (count of specific event type/sum of all event type counts) × 100%.

Table S5. Housekeeping gene alteration patterns and incidence in *H. pylori* mixed infections.

| **PNGAE** | **Pt No.** | ***atpA*** | ***efp*** | ***mutY*** | ***ppa*** | ***trpC*** | ***ureI*** | ***yphC*** |
| --- | --- | --- | --- | --- | --- | --- | --- | --- |
| Single-gene alterations A | h4,h11,h15 | 7.3% (3/41) | 0% | 0% | 0% | 0% | 0% | 0% |
| Single-gene alterations B | h14 | 0% | 0% | 2.4% (1/41) | 0% | 0% | 0% | 0% |
| Single-gene alterations C | h13 | 0% | 0% | 0% | 0% | 0% | 0% | 2.4% (1/41) |
| Three-gene alterations | h1 | 2.4% (1/41) | 2.4% (1/41) | 0% | 0% | 2.4% (1/41) | 0% | 0% |
| Five-gene alterations | h27 | 2.4% (1/41) | 2.4% (1/41) | 0% | 2.4% (1/41) | 2.4% (1/41) | 2.4% (1/41) | 0% |
| Seven-gene alterations | h5,h16,h24,h25 | 9.8% (4/41) | 9.8% (4/41) | 9.8% (4/41) | 9.8% (4/41) | 9.8% (4/41) | 9.8% (4/41) | 9.8% (4/41) |

**Note:** PNGAE patterns based on the number of gene alteration events. Proportion = (count of specific event type/sum of all event type counts) × 100%.

Table S6. Frequency of SDR nullification events (SDR = 0) among genetically unrelated *H. pylori* strains at housekeeping gene loci of clinical isolates.

| **Housekeeping gene (Frequency)** | **SDR nullification events** |
| --- | --- |
| *atpA* (17) | A(H71,H72,H73,H74,H75,H76,H77,H78) vs. A(H113,H114,H115,H116,H121,H122,H123,H124) = 0  C(H79,H80,H81,H82,H83,H84,H85) vs. C(H117,H118,H119, H120) = 0  AD(H57,H58;H65,H66,H68,H69,H70) vs. ACD(H50;H94;H51) = 0  AD(H57,H58;H65,H66,H68,H69,H70) vs. AC(H109;H107) = 0  AD(H57,H58;H65,H66,H68,H69,H70) vs. A(H75) = 0  AD(H57,H58;H65,H66,H68,H69,H70) vs. A(H13) = 0  AD(H57,H58;H65,H66,H68H69,H70) vs. C(H18) = 0  ACD(H50;H94;H51) vs. AC(H109;H107) = 0  ACD(H50;H94;H51) vs. A(H13) = 0  ACD(H50;H94;H51) vs. C(H18) = 0  ACD(H50;H94;H5) vs. A(H75) = 0  AC(H109;H107) vs. (H13) = 0  AC(H109;H107) vs. (H18) = 0  AC(H109;H107) vs. (H75) = 0  A(H13) vs. C(H18) = 0  A(H13) vs. A(H75) = 0  17. C(H18) vs. A(H75) = 0 |
| *efp* (2) | A(H71,H72,H73,H74,H75,H76,H77,H78) vs. A(H113,H114,H115,H116,H121,H122,H123,H124) = 0  2. C(H79,H80,H81,H82,H83,H84,H85) vs. C(H117,H118,H119,H120) = 0 |
| *mutY* (2) | A(H71,H72,H73,H74,H75,H76,H77,H78) vs. A(H113,H114,H115,H116,H121,H122,H123,H124) = 0  2. C(H79,H80,H81,H82,H83,H84,H85) vs. C(H117,H118,H119,H120) = 0 |
| *ppa* (4) | A(H71,H72,H73,H74,H75,H76,H77,H78) vs. A(H113,H114,H115,H116,H121,H122,H123,H124) = 0  C(H79,H80,H81,H82,H83,H84,H85) vs. C(H117,H118,H119,H120) = 0  CD(H14;H15) vs. C(H27,H28,H29,H30,H31,H32,H33) = 0  4. ACD(H20;H19;H18) vs. AD(H55,H56,H57,H58,H59,H60,H61,H62,H97;H63,H64,H65,H66,H67,H68,H69,H70) = 0 |
| *trpC* (3) | A(H71,H72,H73,H74,H75,H76,H77,H78) vs. (H113,H114,H115,H116,H121,H122,H123,H124) = 0  C(H79,H80,H81,H82,H83,H84,H85) vs. C(H117,H118,H119,H120) = 0  3. ACD(H88;H89;H95) vs. ACD(H92;H53;H93) = 0 |
| *ureI* (2) | A(H71,H72,H73,H74,H75,H76,H77,H78) vs. A(H113,H114,H115,H116,H121,H122,H123,H124) = 0  2. C(H79,H80,H81,H82,H83,H84,H85) vs. C(H117,H118,H119,H120) = 0 |
| *yphC* (4) | A(H71,H72,H73,H74,H75,H76,H77,H78) vs. A(H113,H114,H115,H116,H121,H122,H123,H124) = 0  C(H79,H80,H81,H82,H83,H84,H85) vs. C(H117,H118,H119,H120) = 0  C(H42) vs. ACD(H54;H25;H26) = 0  4. D(H49 ) vs. CD(H24;H44) = 0 |

**Note**: NI = number of instances with SDR = 0 between strains from different patients, as shown in Table 3. SDR, sequence difference ratio.

Table S10. Statistical table of SDR values from housekeeping gene *trpC* alignment: *H. pylori* strain H42 vs. patient strain profiles.

| **Pt** | ***trpC*** | **SDR** |  |
| --- | --- | --- | --- |
| h1 | AD(H13;H10) vs.C(H42) | 24.12% |  |
|  | C(H21) vs.C(H42) | 24.34% |  |
| h2 | ACD(H11;H22;H12) vs.C(H42) | 24.34% |  |
| h3 | CD(H14;H15) vs.C(H42) | 25.00% |  |
| h4 | ACD(H20;H18;H19) vs.C(H42) | 25.00% |  |
| h5 | A(H23) vs.C(H42) | 25.66% |  |
| h6 | CD(H24;H44) vs.C(H42) | 23.89% |  |
| h7 | ACD(H54;H25;H26) vs.C(H42) | 29.87% |  |
| h8 | C(H27,H28,H29,H30,H31,H32,H33) vs.C(H42) | 25.00% |  |
| h9 | D(H34,H35,H36,H37,H38,H39,H40,H41) vs.C(H42) | 24.56% |  |
| h10 | ACD(H45;H46,H48;H47) vs.C(H42) | 25.44% |  |
| h11 | AC(H17;H9) vs.C(H42) | 25.22% |  |
| h12 | ACD(H50;H94;H51) vs.C(H42) | 26.33% |  |
| h13 | ACD(H86;H87;H52) vs.C(H42) | 24.56% |  |
| h14 | ACD(H92;H53;H93) vs.C(H42) | 25.00% |  |
| h15 | AD(H55,H56,H57,H58,H59,H60,H61,H62,H97;H63,H64,H65,H66,H67,H68,H69,H70) vs.C(H42) | 25.00% |  |
| h16 | A(H71,H72,H73,H74,H75,H76,H77,H78) vs.C(H42) | 24.78% |  |
|  | C(H79,H80,H81,H82,H83,H84,H85) vs.C(H42) | 25.00% |  |
| h17 | ACD(H88;H89;H95) vs.C(H42) | 25.00% |  |
| h18 | ACD(H101;H90;H91) vs.C(H42) | 25.44% |  |
| h19 | ACD(H96;H98;H99) vs.C(H42) | 25.66% |  |
| h20 | ACD(H100;H102;H103) vs.C(H42) | 24.56% |  |
| h21 | AC(H104;H105) vs.C(H42) | 25.22% |  |
| h22 | CD(H108;H106) vs.C(H42) | 24.78% |  |
| h23 | AC(H109;H107) vs.C(H42) | 24.78% |  |
| h24 | A(H113,H114,H115,H116,H121,H122,H123,H124) vs C(H42) | 24.78% |  |
|  | C(H117,H118,H119,H120) vs.C(H42) | 25.00% |  |
| h25 | AD(H125,H126,H127,H128,H129,H130,H131,H132;H133,H134,H135,H136,H137,H138) vs.C(H42) | 23.67% |  |
|  | C(H139) vs.C(H42) | 25.66% |  |
|  | C(H140) vs.C(H42) | 24.34% |  |
| h26 | A(H141,H142,H143,H144,H145) vs.C(H42) | 25.22% |  |
| h27 | A(H156) vs.C(H42) | 24.78% |  |
|  | AC(H146,H147,H148,H149,H155,H157,H158,H159,H160;H150,H151,H152,H153,H154,H161,H162,H163,H164) vs.C(H42) | 24.78% |  |
| h28 | D(H49) vs.C(H42) | 24.56% |  |
| CS | 34 | |  |
| TC | 2 | |  |
| CO | 32 | | |
| Max | 29.87% | | |
| Min | 23.67% | | |
| *p*5 | 23.89% | | |
| *p*95 | 25.66% | | |
| *p** | 0.000 | | |
| RR | 23.89–25.66% | | |

**Note:** CS, count of sequence alignments between the *trpC* gene of strain H42 and those in each patient’s profile within the group; TC, total number of profiles with increased gene locus mutations in each patient with *H. pylori* mixed infection; CO (count of observations for the group’s profiles) = CS − TC; *p**, *p*-value of the Shapiro-Wilk (S-W) normality test; RR, reference ranges of SDR values for the group. For additional details, please refer to the footnotes of Supplementary Table 7. SDR, sequence difference ratio; Pt, patient.

# 2 Supplementary Text

**Supplementary Text for Figure 4. Phylogeny of *H. pylori* flagellar genes.**

(a) vs. (b) = sbl: short branch length, indicating a close genetic relationship. (a) vs. (b) = lbl: long branch length indicating a distant genetic relationship. U = genetic unrelatedness; M = genetic mutations; CN = common node (i.e., instances where SDR = 0).

**(A)**

(1,2)VS(3,4) = lbl(Pt.1, U);

(97,98,101,5) (Pt.2);

(12,13)VS(14,15) = lbl(Pt.5, U);

(16,17,18)(Pt.6);

(19,20,21,22)(Pt.7);

(23,24,25,26)(Pt.8);

(27,28,29,30)(Pt.9);

(31,32,33,34)(Pt.10);

(35,36,37,38)(Pt.11);

(47)VS(104) = sbl(Pt.15, M);

(48)VS(49) = sbl(Pt.16, M);

(50,51,52;57)(Pt.17);

(63)VS(64,65,66) = lbl(Pt.21, U);

(70,71,72,96)(Pt.22);

(73,74,75,76)(Pt.23);

(81,82,83,84)(Pt.25);

(85)VS(86) = sbl(Pt.26, M);

(63)(Pt.21) VS (39, 40) (Pt.12) = CN

**(B)**

(1)VS(2) = sbl(Pt.1, M), (1)VS(3,4) = lbl(Pt.1,U), (2)VS(3,4) = lbl(Pt.1, U);

(97,98,101,5)(Pt.2);

(12,13)VS(14,15) = lbl(Pt.5, U);

(16,17,18)(Pt.6);

(19,20,21,22)(Pt.7);

(23,24,25,26)(Pt.8);

(27,28,29,30)(Pt.9);

(31,32,33,34)(Pt.10);

(35,36,37,38)(Pt.11);

(47,104)(Pt.15);

(48,49)(Pt.16);

(50,51,52;57)(Pt.17);

(63,64,65,66)(Pt.21);

(70,71,72,96)(Pt.22);

(73,74,75,76)(Pt.23);

(81,82,83,84)(Pt.25);

(85,86)(Pt.26).

**(C)**

(1)VS(2) = sbl(Pt.1, M), (1)VS(3,4) = lbl(Pt.1, U), (2)VS(3,4) = sbl(Pt.1, M);

(97,98)VS(101,5) = sbl(Pt.2, M);

(12,13)VS(14,15) = lbl(Pt.5, U);

(16,17,18)(Pt.6);

(19,20,21,22)(Pt.7);

(23,24,25,26)(Pt.8);

(28)VS(27,29,30) = sbl(Pt.9, M);

(31,32)VS(33,34) = sbl(Pt.10, M);

(36)VS(35,37,38) = sbl(Pt.11, M);

(47,104)(Pt.15);

(48,49)(Pt.16);

(50,51,52;57)(Pt.17);

(63,64,65,66)(Pt.21);

(96)VS(70,71,72) = sbl(Pt.22, M);

(73,74,75,76)(Pt.23);

(81,82,83,84)(Pt.25);

(85,86)(Pt.26).

**(D)**

(1,2)VS(3,4) = lbl(Pt.1, U);

(97,98)VS(101,5) = sbl(Pt.2, M)

(12)VS(13) = lbl(U)(Pt.5, U),

(14)VS(15) = sbl(Pt.5, M);

(12)VS(14) = lbl(Pt.5, U), (12)VS(15) = lbl(Pt.5, U), (13)VS(14) = lbl(Pt.5, U), (13)VS(15) = lbl(Pt.5, U);

(16,17)VS(18) = sbl(Pt.6, M);

(19,20,21)VS(22) = sbl(Pt.7, M);

(23)VS(24,25,26) = sbl(Pt.8, M);

(27,29,30)vs(28) = sbl(Pt.9);

(31,32,33,34)(Pt.10);

(35,36,37,38)(Pt.11);

(47,104)(Pt.15);

(48,49)(Pt.16);

(50,51,52;57)(Pt.17);

(63,64,65,66)(Pt.21);

(70,71,72)VS(96) = sbl(Pt.22, M);

(73,74)vs(75) = sbl(Pt.23, M), (73,74)vs(76) = sbl(Pt.23, M), (76)vs(75) = sbl(Pt.23, M);

(81,82,83,84)(Pt.25);

(85,86)(Pt.26).

**(E)**

(1,2)VS(3,4) = lbl(Pt.1, U);

(97,98,101,5)(Pt.2);

(12,13)VS(14,15) = lbl(Pt.5, U);

(16,17,18)(Pt. 6);

(19,20,21,22)(Pt.7);

(23,24,25,26)(Pt.8);

(27,28,29,30)(Pt.9);

(31,32,33,34)(Pt.10);

(35,36,37,38)(Pt.11);

(47,104)(Pt.15);

(48,49)(Pt.16);

(50,51,52;57)(Pt.17);

(63,64,65,66)(Pt.21);

(70,71,72,96)(Pt.22);

(73,74,75,76)(Pt.23);

(81,82,83)VS(84) = sbl(Pt.25, M);

(85,86)(Pt.26).

**(F)**

(1,2)VS(3,4) = lbl(Pt.1, U);

(97,98,101,5)(Pt.2);

(12,13)VS(14,15) = lbl(Pt.5, U);

(18)VS(16,17) = sbl(Pt.6, M);

(19,20,21,22)(Pt.7);

(23,24,25,26)(Pt.8);

(27,28,29,30)(Pt.9);

(31,32)VS(33,34) = sbl(Pt.10, M);

(35,36,37,38)(Pt.11);

(47)VS(104) = sbl(Pt.15, M);

(48,49)(Pt.16);

(51)VS(50,52;57) = sbl(Pt.17, M);

(63,64,65,66)(Pt.21);

(70,71,72)VS(96) = sbl(Pt.22, M);

(73,74,75,76)(Pt.23);

(81,82,83,84)(Pt.25);

(85,86)(Pt.26).

**Supplementary Text for Figure 5. Phylogeny of *H. pylori* housekeeping genes.**

(a) vs. (b) = sbl: short branch length, indicating a close genetic relationship. (a) vs. b) = lbl: long branch length indicating a distant genetic relationship. U = genetic unrelatedness, M = genetic mutations, CN = common node (i.e., instances where SDR = zero).

**(A)**

(H13) VS (H21,H10) = lbl(Pt.h1, U);

(H20,H19)VS(H18) = lbl(Pt.h4, U);

(H23)VS(H42) = lbl(Pt.h5, U);

(H17)VS(H9) = sbl(Pt. h11, M);

(H86,H87,H52)(Pt.h13);

(H92,H53,H93) (Pt.h14);

(H55,H56,H59-H64,H97)VS(H57,H58,H65,H66,H68-H70) = lbl(Pt.h15, U);

(H75)VS(H79-H85) = lbl(Pt.h16, U), (H71-H78)VS(H79-H85) = lbl(Pt. h16, U), (H75)VS(H71-H78) = lbl(Pt. h16, U);

(H113-H116,H121-H124)VS(H117-H120) = lbl(Pt. h24, U);

(H125-H138)VS(H139) = lbl(Pt. h25, U), (H125-H138)VS(H140) = lbl(Pt. h25, U),(H139)VS(H140) = lbl(Pt.h25, U);

(H156)VS(H160) = sbl(Pt.h27, M), (H156)VS(H146-H155,H157-H159,H161-H164) = sbl(Pt.h27, M),

(H160)VS(H146-H155,H157-H159,H161-H164) = sbl(Pt.h27, M);

(H117-H120)(Pt.h24)VS(H79-H85)(Pt.h16) = CN;

(H52,H86,H87)(Pt.h13)VS(H71-H78)(Pt.h16)VS (H113-H116,H121-H124)(Pt. h24) = CN;

(H50,H51,H94)(Pt.h12)VS(H57,H58,H65,H66,H68-H70)(Pt.h15)VS(H107,H109)(Pt.h23)VS(H13)(Pt.h1)VS(H18)(Pt.h4)VS(H75)(Pt.h16) = CN.

**(B)**

(H13) VS (H21,H10) = sbl(Pt.h1, M);

(H20,H18,H19)(Pt.h4);

(H23)VS(H42) = lbl(Pt.h5, U);

(H17,H9)(Pt.h11);

(H86,H87,H52)(Pt. h13);

(H92,H53,H93) (Pt. h14);

(H55-H70,H97)(Pt. h15);

(H71-H78)VS(H79-H85) = lbl(Pt. h16, U);

(H113-H116,H121-H124)VS(H117-H120) = lbl(Pt. h24, U);

(H125-H138)VS(H139) = lbl(Pt. h25, U), (H125-H138)VS(H140) = lbl(Pt. h25, U), (H139)VS(H140) = lbl(Pt.h25, U);

(H156)VS(H146-H149,H151,H155,H157-H160,H162,H163) = sbl(Pt.h27, M), (H156)VS(H150,H152-H154,H161,H164) = sbl(Pt.h27, M), (H146-H149,H151,H155,H157-H160,H162,H163)VS(H150,H152-H154,H161,H164) = sbl(Pt.h27, M);

(H117-H120)(Pt.h24)VS(H79-H85)(Pt.h16) = CN;

(H71-H78)(Pt.h16)VS(H113-H116,H121-H124)(Pt.h24) = CN.

**(C)**

(H13,H21,H10)(Pt.h1);

(H20,H18,H19)(Pt.h4);

(H23)VS(H42) = lbl(Pt.h5, U);

(H17,H9)(Pt.h11);

(H86,H87,H52)(Pt.h13);

(H92,H53)VS(H93) = sbl(Pt.h14, M);

(H55-H70,H97)(Pt. h15);

(H71-H78)VS(H79-H85) = lbl(Pt.h16, U);

(H113-H116,H121-H124)VS(H117-H120) = lbl(Pt.h24, U);

(H125-H138)VS(H139) = lbl(Pt. h25, U), (H125-H138)VS(H140) = lbl(Pt.h25, U);

(H139)VS(H140) = sbl(Pt.h25,M);(H146-H164)(Pt.h27);

(H117-H120)(Pt.h24)VS(H79-H85)(Pt.h16) = CN;

(H71-H78)(Pt.h16)VS(H113-H116,H121-H124) (Pt.h24) = CN.

**(D)**

(H13,H21,H10)(Pt.h1);

(H20,H18,H19)(Pt.h4);

(H23)VS(H42) = lbl(Pt.h5, U);

(H17,H9)(Pt. h11);

(H86,H87,H52)(Pt. h13);

(H92,H53,H93) (Pt. h14);

(H55-H70,H97)(Pt. h15);

(H71-H78)VS(H79-H85) = lbl(Pt. h16, U);

(H113-H116,H121-H124)VS(H117-H120) = lbl(Pt. h24, U);

(H125-H138)VS(H139) = lbl(Pt. h25, U), (H125-H138)VS(H140) = lbl(Pt. h25, U), (H139)VS(H140) = lbl(Pt.h25, U);

(H156)VS(H146-H155,H157-H164) = lbl(Pt.h27, M);

(H55-H70,H97)(Pt. h15)VS(H20,H18,H19)(Pt.h4) = CN;

(H117-H120)(Pt.h24)VS(H79-H85)(Pt.h16) = CN;

(H71-H78)(Pt.h16)VS(H113-H116,H121-H124)(Pt.h24) = CN.

**(E)**

(H13;H10)VS(H21) = sbl(Pt.h1, M);

(H20,H18,H19)(Pt.h4);

(H23)VS(H42) = lbl(Pt.h5, U);

(H17,H9)(Pt. h11);

(H86,H87,H52)(Pt. h13);

(H92,H53,H93) (Pt. h14);

(H55-H70,H97)(Pt. h15);

(H71-H78)VS(H79-H85) = lbl(Pt. h16, U);

(H113-H116,H121-H124)VS(H117-H120) = lbl(Pt. h24, U);

(H125-H138)VS(H139) = lbl(Pt. h25, U), (H125-H138)VS(H140) = lbl(Pt. h25, U), (H139)VS(H140) = lbl(Pt.h25, U);

(H156)VS(H146-H155,H157-H164) = sbl(Pt.h27, M);

(H117-H120)(Pt.h24)VS(H79-H85)(Pt.h16) =CN;

(H71-H78)(Pt.h16)VS(H113-H116,H121-H124) (Pt.h24) = CN;

(H92,H53,H93) (Pt. h14)VS(H88,H89,H95)(Pt. h17) = CN.

**(F)**

(H13,H21,H10)(Pt.h1);

(H20,H18,H19)(Pt.h4);

(H23)VS(H42) = lbl(Pt.h5, U);

(H17,H9)(Pt.h11);

(H86,H87,H52)(Pt.h13);

(H92,H53,H93) (Pt.h14);

(H55-H70,H97)(Pt.h15);

(H71-H78)VS(H79-H85) = lbl(Pt.h16, U);

(H113-H116,H121-H124)VS(H117-H120) = lbl(Pt.h24, U);

(H125-H138)VS(H139) = lbl(Pt.h25, U), (H125-H138)VS(H140) = lbl(Pt.h25, U), (H139)VS(H140) = lbl(Pt.h25, U);

(H147,H156)VS(H146,H148-H155,H157-H164) = sbl(Pt.h27, M);

(H117-H120)(Pt.h24)VS(H79-H85)(Pt.h16) = CN;

(H71-H78)(Pt.h16)VS(H113-H116,H121-H124) (Pt.h24) = CN.

**(G)**

(H13,H21,H10)(Pt.h1);

(H20,H18,H19)(Pt.h4);

(H23)VS(H42) = lbl(Pt.h5, U);

(H17,H9)(Pt. h11);

(H86)VS(H87,H52) = lbl(Pt.h13, U);

(H92,H53,H93) (Pt.h14);

(H55-H70,H97)(Pt.h15);

(H71-H78)VS(H79-H85) = lbl(Pt.h16, U);

(H113-H116),(H121-H124)VS(H117-H120) = lbl(Pt.h24, U);

(H125-H138)VS(H139) = lbl(Pt.h25, U), (H125-H138)VS(H140) = lbl(Pt.h25, U), (H139)VS(H140) = lbl(Pt.h25, U);

(H146-H164)(Pt.h27);

(H117-H120)(Pt.h24)VS(H79-H85)(Pt.h16) = CN;

(H71-H78)(Pt.h16)VS(H113-H116,H121-H124) (Pt.h24) = CN;

(H42)(Pt.h5)VS(H54,H25,H26)(Pt.h7) = CN;

(H49)(Pt.h28)VS(H24,H44)(Pt.h6) = CN.
